# Tissue Kallikrein Inhibitors Based on the Sunflower Trypsin Inhibitor Scaffold – A Potential Therapeutic Intervention for Skin Diseases

**DOI:** 10.1371/journal.pone.0166268

**Published:** 2016-11-08

**Authors:** Wenjie Chen, Veronica A. Kinsler, Derek Macmillan, Wei-Li Di

**Affiliations:** 1 Infection, Immunity and Inflammation Programme, Immunobiology Section, UCL GOS Institute of Child Health, London, United Kingdom; 2 Genetics and Genomic Medicine Programme, UCL GOS Institute of Child Health, London, United Kingdom; 3 Department of Chemistry, University College London, London, United Kingdom; Uniwersytet Gdanski, POLAND

## Abstract

Tissue kallikreins (KLKs), in particular KLK5, 7 and 14 are the major serine proteases in the skin responsible for skin shedding and activation of inflammatory cell signaling. In the normal skin, their activities are controlled by an endogenous protein protease inhibitor encoded by the *SPINK5* gene. Loss-of-function mutations in *SPINK5* leads to enhanced skin kallikrein activities and cause the skin disease Netherton Syndrome (NS). We have been developing inhibitors based on the Sunflower Trypsin Inhibitor 1 (SFTI-1) scaffold, a 14 amino acids head-to-tail bicyclic peptide with a disulfide bond. To optimize a previously reported SFTI-1 analogue (I10H), we made five analogues with additional substitutions, two of which showed improved inhibition. We then combined those substitutions and discovered a variant (Analogue 6) that displayed dual inhibition of KLK5 (tryptic) and KLK7 (chymotryptic). Analogue 6 attained a tenfold increase in KLK5 inhibition potency with an Isothermal Titration Calorimetry (ITC) K_d_ of 20nM. Furthermore, it selectively inhibits KLK5 and KLK14 over seven other serine proteases. Its biological function was ascertained by full suppression of KLK5-induced Protease-Activated Receptor 2 (PAR-2) dependent intracellular calcium mobilization and postponement of Interleukin-8 (IL-8) secretion in cell model. Moreover, Analogue 6 permeates through the cornified layer of *in vitro* organotypic skin equivalent culture and inhibits protease activities therein, providing a potential drug lead for the treatment of NS.

## Introduction

The Stratum Corneum forms the outermost layer of human skin and is composed of non-viable flattened corneocytes stacked in multiple layers. With the extracellular space filled by lipids, connected to insoluble cross-linked proteins underneath the plasma membrane, it becomes an effective barrier to many substances including water. Adjacent corneocytes are further connected by the cell-cell adhesion complex known as corneodesmosomes, comprising specialized proteins such as Desmoglein 1 and Desmocollin 1 [1]. In the normal skin, proteolysis of those structural proteins results in breakdown of corneodesmosomes leading to corneocyte detachment and controlled skin shedding *i*.*e*. desquamation [2]. The secreted proteases responsible for corneodesmosome degradation are Kallikrein 5 (KLK5) and Kallikrein 7 (KLK7) [3]. These are serine proteases in the 15-membered human tissue kallikreins family of which KLK5 is tryptic and KLK7 is chymotryptic. Together with KLK14, these skin kallikreins participate in a proteolytic cascade and play a major role in desquamation [4]. Moreover, KLK5 and KLK14 have been implicated in skin inflammation by activating the PAR-2 pathway leading to release of inflammatory cytokines including IL-8 [5–7].

Activity of the aforementioned kallikreins is specifically controlled by their endogenous inhibitor Lympho-Epithelial Kazal-Type related Inhibitor (LEKTI), encoded by the gene *SPINK5* [8]. Loss-of-function mutations in *SPINK5* cause the rare genetic skin disease Netherton Syndrome (NS) [9]. As a result of unregulated skin kallikreins activity and excessive skin desquamation, NS patients suffer from scaly skin, atopic manifestation, growth retardation and dehydration, which can be lethal for infants. There is no approved drug hitherto to specifically treat NS. Treatment is currently symptomatic only, based on emollients and topical steroids, with reports of calcineurin inhibitors [10], immunoglobulin replacement [11], anti-Tumor Necrosis Factor α antibody Infliximab [12], and phototherapy [13]. Most recently, a specific NS treatment based on gene therapy has also been under development [14].

Direct inhibition of skin kallikreins by synthetic inhibitors is an attractive potential therapy for NS as it could target the underlying abnormality. This has led to the development of inhibitors ranging from synthetic LEKTI protein domain D6 [15] for KLK5, small organic molecules [16], depsipeptides [17] and an SFTI-1 analogue [18] for KLK7. Recently, the focus has been on selective multiple kallikrein inhibitors targeting KLK5, KLK7 and KLK14. For example, dual inhibition of KLK5 and KLK7 by isomannide-based peptidomimetics [19] and derivatives of 1,2,4-triazole [20], coumarin-3-carboxylate [21] and benzoxazinone [22] multiple kallikrein inhibitors have been described. Besides these small organics, a set of SFTI-1 analogues with 6 out of its 14 amino acid substituted showed multiple kallikrein inhibition and selectivity against other unrelated serine proteases [23].

KLK5 is a key player in the pathogenesis of NS [24] and an initiator of proteolytic cascade in the skin epidermis as it is able to activate itself, pro-KLK7 and pro-KLK14 [25]. We therefore aimed to develop a specific KLK5 inhibitor by making analogues of the previously reported SFTI-1 analogue I10H [26], which has the isoleucine substituted with histidine on position 10 of the SFTI-1 sequence. Though serine proteases have a substrate specificity binding pocket, it only crudely distinguishes them between trypsin-like, chymotrypsin-like or elastase-like. Their refined substrate specificity probably arises from the additional specific interactions formed outside of the binding pocket over a large surface area. Developing a specific serine protease inhibitor is therefore a similar task to developing protein-protein interaction inhibitors as they often require molecules that form multiple interactions over a large and flexible surface area with the target. This is easily achieved by natural specific protein protease inhibitors but a difficult problem for small organic molecules to solve. Covering large interaction surface area without becoming non-druglike is more amenable to macrocyclic molecules *e*.*g*. SFTI-1.

**Fig 1:**
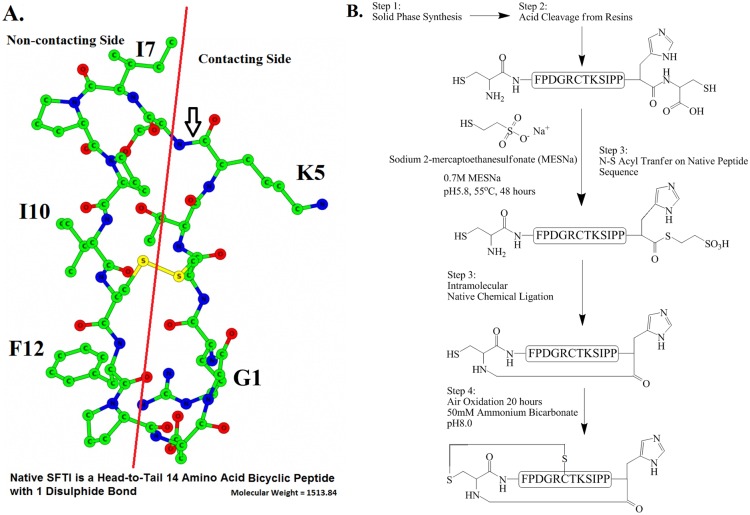
Chemical Structure of Sunflower Trypsin Inhibitor-1 and the “HC” Synthetic Strategy. Chemical structure of native SFTI (**A**) and the synthetic strategy for “HC” analogue synthesis (**B**) are shown. Amino acid residues and their position in the native peptide sequence are labelled in letters and numbers respectively. The P1 residue which will fit into the enzyme substrate binding pocket is lysine located at position 5 with the scissile bond indicated by an arrow. The side of SFTI-1 that forms direct contact with the enzyme and the non-contacting side are segregated by the red line.

There are currently two main strategies for making SFTI-1 (Fig 1, Panel A), for both of which the backbone cyclization step is yield-determining. First is the use of acid-labile resins of which the linear peptide is cyclized while its sidechains remain protected [27]. On-resin cyclization followed by disulfide formation with selective deprotection of the cysteine residues has also been described [28]. We employed the second strategy native chemical ligation involving intramolecular reaction of a peptide thioester with its terminal cysteine [29]. To make the thioester, we exploited the mechanism of N-S acyl transfer on cysteine (Fig 1, Panel B), which offers a convenient reaction setup and does not require special resins or amino acids. This method gives the best yield of SFTI-1 analogues when the cysteine is preceded by a glycine or histidine residue (“HC”) [30]. Our previous results showed that a histidine instead of glycine substitution on position 10 of the native SFTI (SFTI-1) sequence provided a stronger KLK5 inhibitor [26]. We therefore started by making a single substitution (H-GRCTKSIPP**H**CFPD-OH, I10H) and rearrangement of the SFTI-1 sequence H-GRCTKSIPPICFPD-OH to H-CFPDGRCTKSIPP**H**C-OH for thioester formation (Fig 1, Panel B).

The potency of KLK5 inhibition by I10H was first compared to a few reference compounds using a fluorescence-based enzyme assay. I10H is 80 times more potent than p-aminobenzamidine (IC_50_ 0.76 *vs*. 60.45 μM) and retained a similar potency to that of native SFTI. To optimize I10H in terms of potency and selectivity, six more of its analogues were made. Here we report a novel triple substituted SFTI-1 variant (Analogue 6) with bifunctional protease inhibition properties. Analogue 6 is a potent KLK5 inhibitor as determined by enzyme inhibition assay, ITC and *in vitro* cell-based assay. It also displayed multiple kallikrein inhibition with preference for KLK5 and KLK14 over KLK7, KLK8, matriptase and human plasma plasmin. The biological function of Analogue 6 was further verified by its ability to delay KLK5-induced PAR-2 dependent IL-8 release in keratinocytes and inhibit skin proteases in *in vitro* skin equivalent cultures.

## Results

### Design, Synthesis and Proteases Inhibition of I10H Analogues

Inhibition of KLK5 by I10H as well as zinc, p-aminobenzamidine and native SFTI was first tested using the fluorogenic substrate Boc-VPR-AMC (S1 Table and S1 Fig). I10H (IC_50_ 0.76 μM) showed a four and eighty fold potency increase compared to the known KLK5 inhibitor zinc (IC_50_ 2.94 μM) and the unspecific trypsin-like protease inhibitor p-aminobenzamidine (IC_50_ 60.45 μM) respectively. The IC_50_ of zinc is in a comparable range to that of its previously reported K_i_ value [31]. Compared to that of native SFTI (IC_50_ 0.30 μM), the potency is however reduced slightly by twofold. Overall, I10H remained a relatively strong KLK5 inhibitor and so we continued with the development I10H analogues.

**Fig 2:**
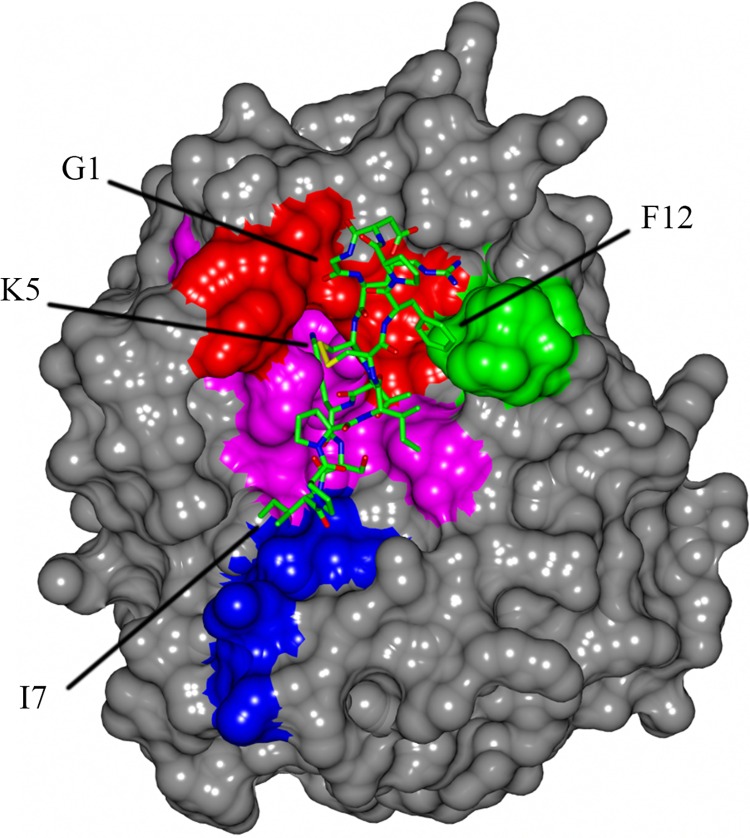
Regions Targeted by I10H Analogues Outside of the KLK5 Substrate Binding Pocket. Molecular docking has revealed several regions (blue, green and red) of KLK5 outside the substrate specificity binding pocket (magenta) that SFTI-1 (shown in cylinders) could engage for extended specific interactions. Surface display of the model was generated by CCP4MG [34].

The crystal structure of KLK5 from the Protein Data Bank (PDB) ID 2PSY [31] was superimposed onto bovine trypsin complexed to native SFTI (PDB ID ISFY) [32] to illustrate potential SFTI-KLK5 interactions (Fig 2). With amino acid sequence alignment between all other kallikrein members and matriptase, a few KLK5 residues that I10H could target specifically were identified (Table 1). The I10H analogues listed in Table 1 were synthesized and tested by fluorogenic assay with Boc-VPR-AMC as the substrate (Table 2). Analogue 1 and 2 showed a significant improvement on KLK5 inhibition compared to that of I10H. Changing the isoleucine to lysine (Analogue 5) severely hampered KLK5 inhibition. Although Analogues 3 and 4 were not stronger KLK5 inhibitors, our synthetic strategy consistently gave a final yield of around 20%.

**Table 1:** Design of I10H Analogues.

KLK5 Motifs ^a^	I10H Analogues ^b^	Modification	Potential Interactions
**H**PGHS (green region Fig 2)	GRCTKSIPPHC**Y**PD	F→Y (F12Y)	hydrogen bond between hydroxyl of tyrosine and nitrogen of histidine
**H**PGHS (green region Fig 2)	GRCTKSIPPHC**W**PD	F→W (F12W)	π-π stacking between tryptophan and histidine or hydrogen-π interaction
P**N**QLY (blue region Fig 2)	GRCTKS**K**PPHCFPD	I→K (I7K)	hydrogen bond between NH of lysine and C = O of asparagine side chain
SWG**D**YP (red region Fig 2)	**N**RCTKSIPPHCFPD	G→N (G1N)	hydrogen bond between C = O of aspartate and NH of asparagine side chain
The Substrate Pocket (magenta region Fig 2)	GRCT**R**SIPPHCFPD	K→R (K5R)	Substrate binding pocket of KLK5 is predicted to have preference for arginine [33]

^*a*^ Letters in bold are the amino acid residues that were expected to form the interactions.

^*b*^ Letters in bold are the additional substitutions on the I10H sequence.

**Table 2:** I10H Analogues Synthetic Yields and Their KLK5 Inhibition.

Analogues	Sequence ^a^	Molecular Weight	Isolated yield from linear precursor /%	Boc-VPR-AMC IC_50_ (N = 3)/nM ^b^	Inhibitory Constant (K_i_)/nM
I10H	GRCTKSIPPHCFPD	1537.8 (calculated) 1538.3 (measured)	19	352 ±144	322
1 (K5R_I10H)	GRCT**R**SIPPHCFPD	1565.8 (calculated) 1566.2 (measured)	24	65 ±10	60
2 (I10H_F12W)	GRCTKSIPPHC**W**PD	1576.8 (calculated) 1577.2 (measured)	25	36 ±15	33
3 (G1N_I10H)	**N**RCTKSIPPHCFPD	1594.8 (calculated) 1595.2 (measured)	25	511 ±86	467
4 (I10H_F12Y)	GRCTKSIPPHC**Y**PD	1553.8 (calculated) 1554.1 (measured)	19	334 ±31	305
5 (I7K_I10H)	GRCTKS**K**PPHCFPD	1552.8 (calculated) 1553.2 (measured)	16 ^c^	>10,000	>9000

^*a*^ Letters in bold indicate the additional substitution made to I10H.

^*b*^ IC_50_ curves of each tested ligand are displayed in S2 Fig.

^*c*^ reduced yield due to repeated purification.

Based on the results in Table 2, a sixth analogue (Analogue 6) incorporating the K5R and F12W substitution (GRCT**R**SIPPHC**W**PD) was made. The synthesis again offered a final yield of 24% giving us 10.8 mg of the pure inhibitor from 46.7 mg of linear peptide. The inhibition of this triple substituted SFTI-1 variant along with its predecessors (I10H and Analogue 2) and native SFTI were tested on ten serine proteases (Table 3), seven trypsin-like, two elastase and one chymotrypsin-like (KLK7). Human neutrophil elastase and porcine pancreatic elastase 1 were not inhibited by any of the SFTI-1 analogues. Human plasma kallikrein was only inhibited slightly by Analogue 6 at 10 μM.

**Table 3:** Enzyme Inhibitory Profile of I10H Analogues.

Protease ^a^*/nM*	Substrate ^a^*/μM*	IC_50_ (N = 3) /nM ^b^				
		Native SFTI	I10H	Analogue 1	Analogue 2	Analogue 6
Bovine Trypsin (10)	Boc-VPR-AMC (21)	19 ±1	46 ±2	n.d.	20 ±0.2	23 ±1
KLK14 (10)	Boc-VPR-AMC (45)	30 ±7	470 ±47	n.d.	85 ±6	24 ±2
KLK5 (10)	Boc-VPR-AMC (42)	230 ±3 (K_i_ = 210)	523 ±11 (K_i_ = 478)	n.d.	100 ±2 (K_i_ = 91)	56 ±0.5 (K_i_ = 51)
KLK8 (9)	Boc-VPR-AMC (42)	1286 ±251	1683 ±51	n.d.	563 ±14	373 ±31
Matriptase (10)	Boc-VPR-AMC (21)	1254 ±57	2244 ±567	n.d.	206 ±10	381 ±12
Human Plasma Plasmin (24)	Boc-VPR-AMC (42)	24 ±4	161 ±6	n.d.	25 ±1	407 ±8
KLK7 (10)	BODIPY_FL (Casein 6.5 μg/mL)	18% inhibition at 11 μM	0% inhibition at 11 μM	2289 ±68	28% inhibition at 11 μM	534 ±29
Human Neutrophil Elastase (10)	MeOSuc-AAPV-AMC (41)	0% inhibition at 10 μM	0% inhibition at 10 μM	n.d.	0% inhibition at 10 μM	0% inhibition at 10 μM
Porcine Pancreatic Elastase 1 (46)	MeOSuc-AAPV-AMC (41)	0% inhibition at 10 μM	0% inhibition at 10 μM	n.d.	0% inhibition at 10 μM	0% inhibition at 10 μM
Human Plasma Kallikrein (14)	Boc-VPR-AMC (42)	0% inhibition at 10 μM	0% inhibition at 10 μM	n.d.	0% inhibition at 10 μM	15% inhibition at 10 μM

^*a*^ value denoted in bracket is the final assay concentration.

^*b*^ Fitted IC_50_ curves (except KLK5) of each tested ligand are displayed in S3 Fig.

n.d.: Not Determined.

Natural selective protease inhibition is illustrated by the inhibitory profile of native SFTI (Table 3). Native SFTI inhibited most strongly on bovine trypsin, KLK14 and plasma plasmin over KLK5, KLK8 and matriptase. It is a very poor KLK7 inhibitor and was unable to inhibit plasma kallikrein and the two elastases. Changing the isoleucine at position 10 to histidine on the SFTI-1 sequence not only reduced inhibition of KLK5 by two folds, but to different extents, for all the tested tryptic and chymotryptic proteases. The I10H substitution was most intolerable to KLK14 with an almost 20 fold potency drop compared to native SFTI. In contrast, the additional F12W substitution (Analogue 2) salvaged the adverse effect of I10H and afforded a better enzyme inhibitor.

**Fig 3:**
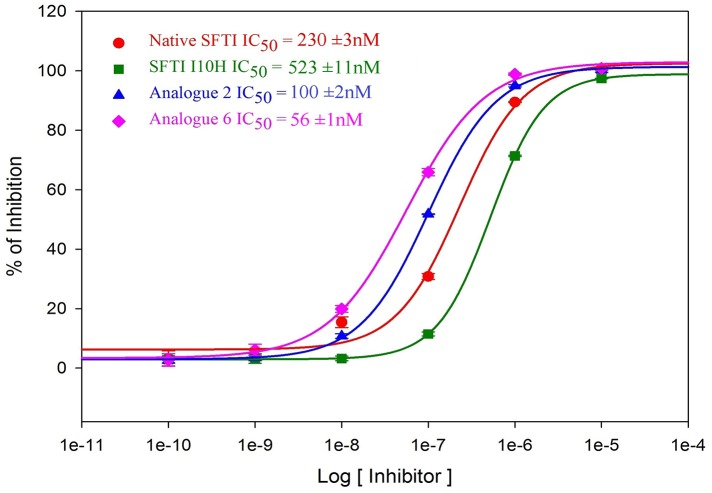
Fitted IC_50_ Curves of KLK5 Inhibition by Native SFTI and Its Analogues. The fitted KLK5 inhibition IC_50_ curves including error bars (standard deviation) of each repeated reading (N = 3) for SFTI-1 and its analogue are shown.

With the third substitution K5R (Analogue 6), a KLK5 inhibitor ten times more potent than I10H (Fig 3) was attained. Analogue 6 is also a strong inhibitor for bovine trypsin, and KLK14 but selective against the other seven tested proteases. Interestingly, Analogue 6 displayed chymotryptic inhibition against KLK7 with a potency near to that of its inhibition on the other tryptic enzymes. This is a major improvement as the binding affinity of native SFTI towards chymotrypsin is of around four orders of magnitude weaker than trypsin [35].

### Dissociation Constants (K_d_) by Isothermal Titration Calorimetry

**Fig 4:**
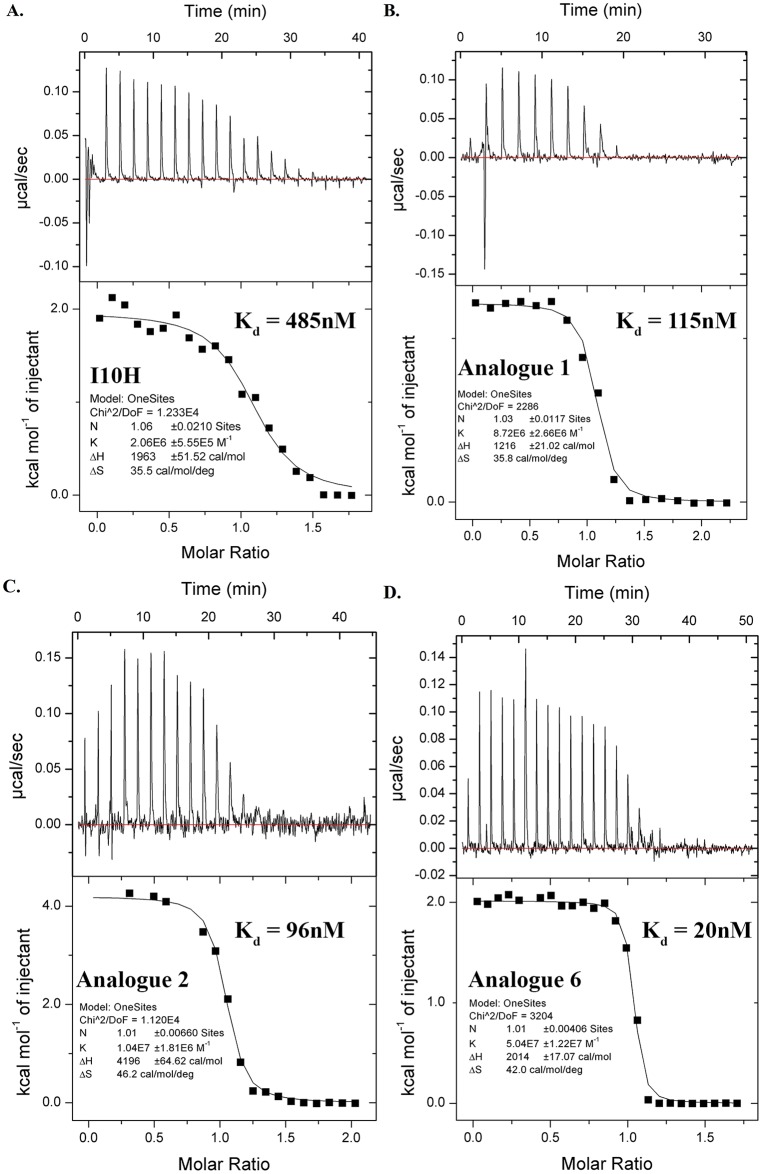
Fitted Isotherms for Binding of I10H and Its Analogues to KLK5 in 1xPBS at 25°C. Fitted isotherms of I10H (A), Analogue 1 (B), Analogue 2 (C) and Analogue 6 (D) are shown. Binding of all tested cyclic peptides to KLK5 are endothermic and entropically driven.

In addition to enzyme assays, we determined the binding affinity (K_d_) and characterized the thermodynamic binding profile (S2 Table) of I10H, Analogue 1, 2 and 6 for KLK5. Their isotherms demonstrated the binding of these cyclic peptides to KLK5 was endothermic and entropically driven (Fig 4). The binding stoichiometry is in agreement with the literature, which was found to be approximately 1:1. In good agreement with the K_i_ values from Table 3, the determined ITC K_d_ for I10H, Analogue 1, 2, and 6 are 485 nM (K_i_ = 478 nM), 115nM, 96 nM (K_i_ = 91 nM) and 20 nM (K_i_ = 51 nM) respectively. The tenfold improved potency from I10H to Analogue 6 in the enzyme inhibition assay is depicted by an increase in binding affinity of around 25 times. These binding constants were determined in phosphate buffered saline (PBS) solution (pH 8.0) at 25°C. However, when tested at the same temperature and similar ionic strength but a different pH (pH 5.5) buffered using 2-(N-morpholino) ethanesulfonic acid (MES), no enthalpy change (ΔH) was observed (data not shown). Unless ΔH was inherently equal to zero under such condition, these SFTI-1 analogues do not bind to KLK5 at pH 5.5.

### Prevention of KLK5-Induced Intracellular Calcium Mobilization

As well as being in the G-protein Coupled Receptor family, PAR-2 is naturally activated by the tethered ligand mechanism [36]. It has been demonstrated that PAR-2 can be activated by KLK5 in keratinocytes [5]. One of the events that take place upon PAR-2 activation is intracellular calcium mobilization. This can be measured by fluorogenic calcium-binding dyes, as the fluorescence increases during calcium release and drops back to base level due to re-uptake. The potential biological activity of SFTI-1 and its analogues were further ascertained by its ability to block KLK5-induced PAR-2 activation in the keratinocyte cell line N-tert. To ensure that the PAR-2 activated intracellular calcium mobilization apparatus was operational in the cell line, the cells were first challenged with the PAR-2 agonist peptide H-SLIGKV-NH_2_ (S4 Fig).

**Fig 5:**
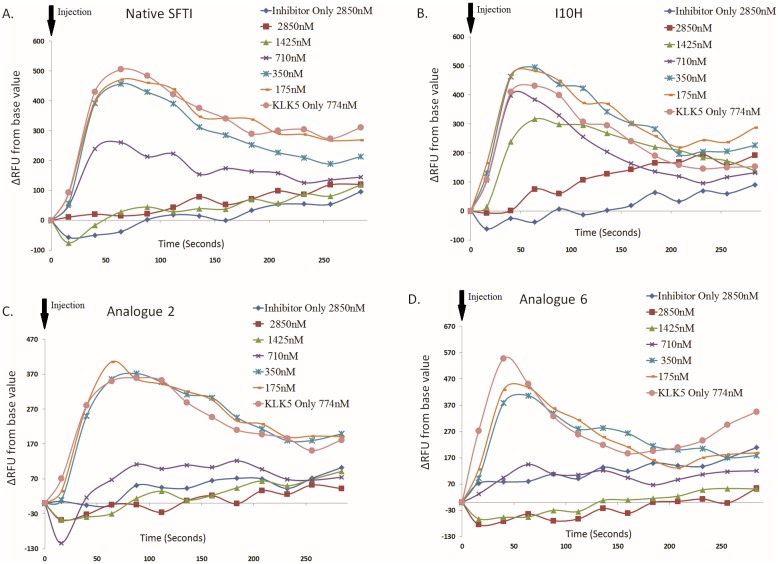
Suppression of KLK5-Induced Intracellular Calcium Mobilization by Native SFTI and Its Analogues. Change in relative fluorescence unit (ΔRFU) was monitored continuously for about 5 minutes after injection of a mixture of KLK5 (774 nM) with various concentrations of native SFTI or its analogues. KLK5 and the inhibitors were diluted in 1xPBS and the concentration values indicate their final concentration in the well. For each tested ligand, a positive control (KLK5 Only) and negative control (Inhibitor Only) were performed to ensure the inhibitor and the diluent does not cause a signal. Total suppression of KLK5-induced intracellular calcium mobilization was achieved at an inhibitor-to-enzyme ratio of 2:1 for native SFTI (A), 4:1 for I10H (B.) and 1:1 for Analogue 2 (C) and Analogue 6 (D).

The cells were then challenged with KLK5 alone and a fluorescence peak was also observed (KLK5 Only, Fig 5). The KLK5 peak had similar peak time to that of the PAR-2 agonist, but returned to background much more slowly. When KLK5 was injected in a mixture containing SFTI-1 or its analogues to the cells, PAR-2 activation was clearly attenuated. Complete suppression of intracellular calcium mobilization was achieved at inhibitor-to-enzyme molar ratio of around 4:1, 2:1 and 1:1 for I10H, native SFTI, and Analogue 2 or 6 respectively (Fig 5). These molar ratios are corroboratory to their comparative IC_50_ values in Table 3 obtained from enzyme inhibition assay.

### Suppression of KLK5-Induced IL-8 Release

**Fig 6:**
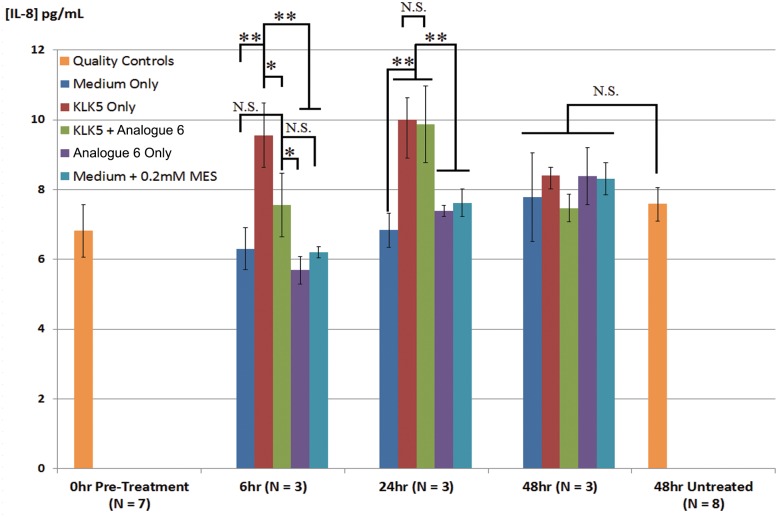
Control of IL-8 Secretion by Analogue 6 in KLK5 Simulated N-tert Skin Cells. Changes in IL-8 level in the cell medium was monitored 6, 24 and 48 hours after identical cell populations were stimulated with fresh medium (Medium Only), or KLK5 (KLK5 Only, f/c 300 nM), or KLK5 + Analogue 6 (KLK5 + Analogue 6), or Analogue 6 (Analogue 6 Only, f/c 1515 nM), or 0.2 mM MES (Medium + 0.2 mM MES). All substances were diluted with cell culture medium. Control samples including the medium with MES were used to ensure that MES which was in the KLK5 protein stock solution did not cause IL-8 level to change. Basal IL-8 level prior to stimulation (0hr pre-treatment) as well as for unstimulated cells 48hrs after (48hr untreated) were also determined to serve as quality controls. A coefficient variation of around 11% and 6% for 0hr pre-treatment and 48hr untreated respectively indicates the status of cell populations in each well was homogenous throughout the experiment. Error bars are standard deviations of IL-8 readings from different wells of cells. N.S. Not Statistically Significant; * p<0.05; **p<0.01.

Although Analogue 6 successfully prevented the immediate effect (intracellular calcium mobilization) of PAR-2 activation, its ability to control the downstream effect of PAR-2 mediated cytokine release remained unknown. It has been shown the production of the cytokine IL-8 is upregulated by PAR-2 activation in different human cell types including keratinocytes [7, 37, 38]. We therefore probed the duration of biological effectiveness of Analogue 6 by determining its ability to control IL-8 secretion in N-tert cells stimulated with KLK5. IL-8 level has indeed increased after KLK5 stimulation in N-tert cells at 6 and 24 hours (p<0.01, Fig 6). The increase was however a transient response as there was no statistical difference found in IL-8 level 48 hours after stimulation between the stimulated and unstimulated cells. At a molar excess of 5:1, Analogue 6 was able to suppress IL-8 increase up to 6 hours (p<0.05) but not 24 hours after stimulation.

### Inhibition of Skin Protease Activity in *in vitro* Skin Model

**Fig 7:**
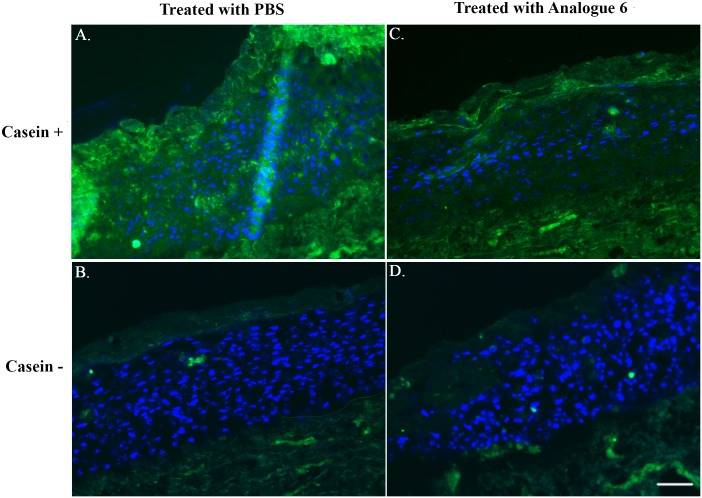
*In-Situ* Zymography in Organotypic Skin Culture (OTC). Fluorescence images from *in-situ* zymography of OTC treated with PBS (A & B) or Analogue 6 (C & D) are shown. For the negative staining, PBS was applied to the cryosection instead of casein substrate solution (B & D). Cell nuclei were counterstained with 4',6-diamidino-2-phenylindole (blue color) and protease activity is signaled by green fluorescence. All images were obtained in MicroManager (Ver.1.4.21) with an exposure time of 1s and images were processed using ImageJ (Ver.1.46) and finalized by Adobe Photoshop CS. Brightness and contrast settings were all the same and applied to the entire images. Scale Bar = 200 μm.

To assess protease inhibition of Analogue 6 in skin-like environment, *in vitro* skin equivalent cultures were generated using primary human keratinocytes and fibroblasts. The matured cultures were topically treated with Analogue 6 (10 μM) in PBS solution once every day for two days. Cryosections of the treated and untreated skin were subjected to *in-situ* zymography with fluorescently labelled casein substrate and examined by fluorescence microscopy. Skin protease activity was shown to be weaker in the epidermis of the treated skin compared to untreated skin (Fig 7). This indicates Analogue 6 has penetrated the cornified layer of the *in vitro* grown skin and inhibited protease activity in the epidermis.

## Discussions and Conclusions

One of the major obstacles for bringing macrocyclic peptide-based drugs to the market is the complexity and cost of manufacturing on a large scale. Here we aimed to develop analogues of SFTI-1 for the potential treatment of the severe rare genetic skin disease Netherton syndrome. We employed the “HC” apparatus by replacing an isoleucine that precedes the cysteine in the native SFTI sequence with a histidine (I10H) to synthesize SFTI-1 analogues. The “HC” apparatus consistently offered a final yield of around 20% on all the analogues we have made in an essentially four-step process (Fig 1, Panel B). The prerequisite of histidine substitution prompted us to first see its effect on biological activity. We showed by means of enzyme assay that I10H retained its protease inhibitory activity albeit is a slightly weaker KLK5 inhibitor than native SFTI.

Equipped with a synthetic strategy (I10H) of good yield without significantly compromising biological activity, we explored a few I10H analogues. The analogue design was based on docking studies and purported for a KLK5 specific inhibitor. Two of the five I10H analogues (Analogue 1 & 2) in Table 2 showed an increased inhibition on KLK5. Analogue 5 suffered a complete loss of activity, which could be the result of taking both isoleucines (I7K_I10H) out of the native SFTI sequence, or the result of the I7K substitution alone. The latter is likely to be true because we have shown that I10H substitution did not significantly alter KLK5 inhibition compared to native SFTI. Moreover, a recent paper showed that KLK5 inhibition was reduced by 90% with the I7K substitution whilst the other isoleucine was kept [39].

By combining substitutions of Analogue 1 and 2, Analogue 6 was made and tested along with native SFTI, I10H, and Analogue 2 on a range of serine proteases (Table 3). None of the substitutions introduced into the native SFTI sequence significantly altered the bovine trypsin inhibitory property of the bicyclic peptide. This was not unexpected because changes such as lysine to arginine and phenylalanine to tryptophan were considered conservative. The most dramatic change was from isoleucine to histidine and I10H was overall the least potent of the four inhibitors for the tested enzymes. It should also be pointed out that the IC_50_ of native SFTI for bovine trypsin is much higher than the previously reported K_i_ values of 0.02–0.1 nM [23, 32]. This is because IC_50_ values are heavily dependent on assay conditions such as the enzyme concentration [E], substrate concentration at half maximal enzymic rate (K_m_), assay substrate concentration [S] and assay temperature. Under optimal assay conditions, IC_50_ is ≈ K_i_ except in the events of tight binding (K_i_ << [E]) and small K_m_ value (K_m_ << [S]). As IC_50_ will never get below [E]/2, appropriate enzyme concentration is paramount for detecting tightly bound ligands. The sudden drop in % of inhibition as inhibitor concentration drops from 100 to 10 nM in S3 Fig (Panel A) indicates bovine trypsin has a high affinity for the substrate Boc-VPR-AMC *i*.*e*. small K_m_. Due to the potential flaws in IC_50_ values, it is a common practice in early stage drug discovery to validate hits by orthogonal assays and determine the true K_d_ of optimized lead molecules using standard techniques such as ITC. Interestingly, a recent report has determined the ITC K_d_ of native SFTI for bovine trypsin to be 7-13nM [40], which underlines the fact that assay values can differ greatly depending on the assay conditions and measurement technique used.

Substitution made on position 10 and 12 of the SFTI-1 sequence has brought universal reduction (I10H) or augmentation (Analogue 2) in enzyme inhibition. As shown in Fig 1 Panel A, only half of the surface of SFTI-1 is in direct contact with the enzyme, and both position 10 and 12 are on the non-contacting side. Changing residues in those positions are likely to have a global effect on the SFTI-1 scaffold, possibly in the forms of ligand rigidity and hydrophobicity. On the other hand, changing residue on position 5 *i*.*e*. the P1 residue [41] in the contacting side will confer selectivity to the ligand. This is commonly known, but the inhibition of plasmin and KLK7 by Analogue 2 and 6 have exemplified that even a minor change such as from lysine to arginine could bring significant selectivity changes.

Not only can Analogue 6 inhibit KLK7, it inhibits KLK5 and 14 more potently than KLK8, matriptase and plasmin. Since the degradation of the corneodesmosome involves the activity of KLK5, KLK7 and KLK14, a multiple kallikrein inhibitor could be the optimal drug for therapy of disease of this process, imitating the endogenous protease LEKTI. This is congruent with a recent patent application [42] that emphasized the importance of multiple protease inhibition (KLK5, 7, 14 and elastase) for NS treatment demonstrated by mice knockouts. Many studies have also shown enhanced expression/activity of KLK5 and 7 in other skin conditions including atopic dermatitis [43, 44], rosacea [45] and psoriasis [46]. The dual inhibition of KLK5 and 7 afforded by Analogue 6 may also be beneficial for those conditions.

It is known that LEKTI, the endogenous skin kallikreins inhibitor is able to inhibit KLK5 (tryptic), KLK7 (chymotryptic) and elastase [47] due to its multiple protein domains. Multiple and simultaneous inhibitions of proteases from different family are considered a privilege of protein protease inhibitors. Another example is the 71 amino acids Bowman-Birk inhibitor (BBI), a bifunctional trypsin-chymotrypsin protein protease inhibitor found in soybean [48]. Its simultaneous, independent and potent inhibition of trypsin and chymotrypsin [49] is achieved through two reactive loops in different regions of the protein. Having a lysine as the P1 residue makes one loop inhibit trypsin and a leucine P1 residue on the other loop confers chymotrypsin inhibition [50]. Though phylogenetically unrelated to BBI [51], SFTI-1 shares strong sequence and structural homology with the BBI reactive loops and is specialized for trypsin inhibition. We have shown here that it is possible to engineer a bifunctional SFTI-1 derived protease inhibitor by substituting the P1 residue lysine to arginine. Although there are previous reports of SFTI-1 variants (containing a P1 arginine) that inhibited KLK5 and KLK7 [23, 39, 52, 53], the dual inhibition property could not be definitively attributed to P1 arginine because multiple substitutions at different positions were also introduced whilst the effect of each individual substitution was not characterized. Due to the small size of SFTI-1, it is however unlikely to afford the simultaneous inhibition of trypsin and chymotrypsin seen with BBI.

We have also demonstrated that the inhibitory effect of substitutions at different positions of a cyclic peptide is additive with the increased inhibition of KLK5 and KLK7 by Analogue 6. For KLK7 inhibition, which is further enhanced by the F12W substitution, the K5R substitution is the primary contributor. However, changing from lysine to arginine is not something one would expect to make a good chymotrypsin inhibitor, though an asparagine residue is found in the substrate specificity pocket of KLK7. Although the fundamental effect of the P1 arginine for dual inhibition remain to be elucidated fully, a trifunctional BBI variant (76 residues) [54] also found in soybean highlights its importance. The variant was able to inhibit trypsin, chymotrypsin and elastase *via* its two inhibitory loops. Favorably, the trypsin-chymotrypsin inhibition was shared on one of the loop and the P1 residue was an arginine.

After a buildup of structure activity relationship in Table 3, we characterized the thermodynamic binding properties of I10H, Analogue 1, 2 and 6 to KLK5 by ITC. Under the test conditions (1xPBS pH 8.0 at 25°C), the binding of all the tested SFTI-1 analogues to KLK5 was endothermic. This is an interesting contrast to the binding of native SFTI with bovine trypsin, which was found to be exothermic [40] albeit the two serine proteases share a highly conserved structural scaffold. A small positive enthalpy (ΔH) but large positive entropy (ΔS) change implies their binding was predominantly entropically driven. Compared to that of I10H, Analogue 2 has a more positive ΔS value but a twofold increase in ΔH. It is likely to have displaced additional water molecules as the larger tryptophan ring comes to close contact with residues in the green region of Fig 2. In contrast, the ΔH of Analogue 1 is halved to that of I10H while the ΔS remained essentially the same. This implies additional bond formation, resulted by a change from lysine to arginine. A plausible scenario is that the guanidine group has formed double hydrogen bonds with the aspartate of KLK5 at the substrate binding pocket (magenta region, Fig 2).

To follow, we tested the ability of native SFTI and its analogues to suppress the proinflammatory PAR-2 signaling activation by KLK5 utilizing a cell-based assay. The assay detects intracellular calcium mobilization, as a result of PAR-2 activation by measuring fluorescence changes. The fluorescence peak induced by KLK5 compared to PAR-2 agonist had a similar peak time at around 40 seconds, but returned to baseline much more slowly. Their slight fluorescence peak shape variation could be explained by the tethered ligand mechanism with regards to the maximum rate of PAR-2 activation. KLK5 activation of PAR-2 is a twostep continuous process involving the cleavage and diffusion of the tethered ligand, but a one-step one-off diffusion process for direct PAR-2 agonist injection.

With regards to the biological function of the SFTI-1 analogues, we further investigated the ability of Analogue 6 to control IL-8 release from N-tert cells stimulated by KLK5 *via* the PAR-2 signaling pathway. At an inhibitor-to-enzyme ratio of 5:1, Analogue 6 was able to delay the KLK5-induced IL-8 release by up to 6 hours but not for 24 hours. The short-lived effect could be a result of selective cellular uptake of Analogue 6 thus allowing the KLK5 to activate PAR-2. The presence of secreted reductase may also break the disulfide bond of Analogue 6, which will make the inhibitor to become more susceptible to protease attacks. Furthermore, we topically applied Analogue 6 to a piece of *in vitro* grown skin and found it has dampened skin protease activity beneath the cornified layer. Although the skin barrier generated from *in vitro* skin equivalent culture is more permeable than normal human skin *in vivo*, the results nevertheless demonstrated the potential efficacy of SFTI-1 derived topical drugs to control skin protease activity.

In summary, we have discovered a novel bifunctional SFTI-1 variant that shows selective and improved inhibition of multiple skin kallikreins. We were able to produce the variant in good yield and its biological effectiveness was validated by a comprehensive follow-up study using biophysical technique, cell-based assay, *in vitro* cell and skin equivalent culture models. This lead could be further developed to mimic the inhibitory fingerprint of LEKTI in the skin for the treatment of NS and potentially more common skin disorders involving skin kallikreins hyperactivity.

## Materials and Methods

### Synthesis of SFTI-1, SFTI I10H, and I10H Analogues

All linear peptide precursors were made by Fast-Moc^™^ chemistry on an ABI433A automated peptide synthesizer. Solid-phase peptide synthesis was performed using standard Fmoc amino acids and pre-loaded Fmoc-Cys (Trt)-NovaSyn^®^TGT resin. With 10 equivalents of each amino acid, coupling reaction was initiated using 0.45 M N,N,N′,N′-Tetramethyl-O-(1H-benzotriazol-1-yl)uronium hexafluorophosphate and 0.45 M 1-Hydroxybenzotriazole hydrate in dimethylformamide. 1.7 equivalent of N,N-diisopropylethylamine in N-methyl-2-pyrrolidone was subsequently added. Fmoc deprotection was achieved by 20% volume to volume (v/v) piperidine in N-methyl-2-pyrrolidone. The assembled peptide was cleaved in v/v 95% trifluoroacetic acid, 2.5% 1, 2-ethanedithiol and 2.5% H_2_O at room temperature with stirring for 4 hours. The resins were then removed by filtration and the peptide in solution was precipitated by diethyl ether. The precipitated peptide was extracted by centrifugation at 2000 g for 15 minutes and then purified by reverse-phase high pressure liquid chromatography (RP-HPLC).

RP-HPLC was performed in Dionex Ultimate 3000 system connected to a Phenomenex Jupiter 10 μm Proteo 90 Å, C12 column (250x21.2 mm). Separations were carried out with a gradient of increasing % of acetonitrile from 5–60% with 0.1% trifluoroacetic acid (v/v). The purified linear peptide was lyophilized, weighted and reconstituted in water. The linear peptide (1 mg/mL) was cyclized in the solution of 0.1 M sodium phosphate buffer (pH 5.8) and 0.7 M sodium 2-mercaptoethane sulfonate. The reaction was left on for 48 hours at 55°C with stirring. The cyclized peptide was purified by RP-HPLC then directly oxidized with addition of ammonium bicarbonate (pH 8.0) at final concentration (f/c) of 50 mM. The oxidized cyclic peptide was lyophilized and re-purified by RP-HPLC. The purified final product was lyophilized and weighted. Quality of each compound was assured by analytical liquid chromatography and mass spectrometry (Waters ACQUITY UPLC SQD) with an ACQUITY BEH C18 column (2.1x50 mm). The mobile phase was an increasing gradient of acetonitrile with 0.1% v/v formic acid. RP-HPLC chromatogram and mass spectrum of each prepared compound are available in S1 File.

### Protease Inhibition Assays

#### General procedures

All assays were performed on 96-well microplates and the change in relative fluorescence unit (RFU) was measured by fluorescence spectrometer (BMG FLUOstar OPTIMA). The excitation/emission wavelength was 360/460 nm for the substrate Boc-VPR-AMC (Bachem AG, Hauptstrasse, Switzerland) and MeOSuc-AAPV-AMC (Bachem AG, Hauptstrasse, Switzerland) and 485/520 nm for BODIPY_FL casein (Invitrogen, Carlsbad, US). Stock solution of Boc-VPR-AMC was prepared by dissolving the solid in dimethyl sulfoxide to 100 mM and 10 mM for MeOSuc-AAPV. For BODIPY_FL casein, it was reconstituted to 1 mg/mL in 1xPBS. Ligand only and enzyme only were served as the negative (100% inhibition, Minimum RFU) and positive (0% inhibition, Maximum RFU) control respectively. The % of inhibition was calculated by the following equation:

$$\%\;\;of\;Inhibition=\left(1-\frac{\left(Sample\;RFU\;-\;Min\;RFU\right)}{\left(Max\;RFU\;\;-\;Min\;RFU\right)}\right)\;X100$$

Each inhibition curves were done in triplicates with a minimum of five concentration points fitted. Fitting of each curve was done using SigmaPlot with the Four Parameter Logistic Function and the IC_50_ calculated. Average of the three calculated IC_50_ values of each tested compound with the standard deviation are used for Tables 2 and 3 and S1 Table. All tested compounds were reconstituted to stock solution in water.

#### Enzyme activations by removal of N-terminal zymogen sequences

Recombinant KLK7, KLK8 and KLK14 (>95% purity by SDS-PAGE, endotoxin <0.1 ng/μg, R&D, Minneapolis, U.S.) stocks were diluted with 1xPBS to a concentration of 120 nM. KLK7 and KLK14 were activated by addition of 1 mM f/c CaCl_2_ (Sigma, Dorset, U.K.), 0.05% v/v Brij35 (Sigma, Dorset, U.K.), 20 nM f/c thermolysin (Sigma, Dorset, U.K.) and incubated at 37°C for 1 hour. The reaction was stopped by addition of ethylenediaminetetraacetic acid (EDTA, 50 mM f/c). KLK8 was activated by addition of 11 nM f/c lysyl endopeptidase (Santa Cruz Biotechnology, Dallas, U.S.) and incubation at 37°C for 0.5 hour. The negative controls (no KLK) for the thermolysin and lysyl endopeptidase activation mix confirmed that the two enzymes do not increase the RFU when mixed with Boc-VPR-AMC. Thermolysin had some residual activity against BODIPY_FL casein accounting for ≈15% of the total cleavage activity. The RFU value reached by the thermolysin control was used as the minimum RFU for calculating the % of inhibition for KLK7.

#### S1 Table

The diluent for all the tested compounds, protein and substrate was 1xPBS pH 8.0. Zinc sulfate and p-aminobenzamidine were purchased from Sigma (Dorset, U.K.). Serially diluted ligands were mixed with 10 nM f/c recombinant KLK5 (>95% purity by SDS-PAGE, endotoxin <0.1 ng/μg, Novoprotein, New Jersey, U.S.). The ligand-protein mixtures were incubated on ice for 15 minutes and 20 μL was transferred to a well of a 96-well microplate (ThermoScientific, Waltham, U.S.) in triplicate. To start the reaction, 100μL of the diluted (41.6 μM f/c) Boc-VPR-AMC substrate solution (contained 0.05% v/v Brij35) pH 8.0 was added. Change in RFU was measured 15 minutes after substrate addition at 25°C.

#### Table 2

The diluent for all the tested compounds, protein and substrate was 1xPBS. Serially diluted ligands were mixed with diluted recombinant KLK5 (10 nM f/c). The ligand-protein mixtures were incubated on ice for one hour and 19.5 μL was pipetted into a well of a 96-well microplate in triplicates. To start the reaction, 100 μL of the diluted (41.6 μM f/c) Boc-VPR-AMC substrate solution (contained 0.05% v/v Brij35) was added. The plate was incubated at 37°C and the RFU reading was taken 15 minutes after.

#### Table 3

The diluent for all the tested compounds, protein and substrate (except KLK7, which was done with 10 mM Tris pH 7.8) was 1xPBS pH 8.0–8.9. The serially diluted ligands were mixed with diluted bovine trypsin (Sigma, Dorset, U.K.) or activated KLK14, or KLK5, or activated KLK8 or matriptase (catalytic domain only, >90% purity by SDS-PAGE, endotoxin <0.1 ng/μg, R&D, Minneapolis, U.S.) or activated KLK7 or human neutrophil elastase (purified from human plasma, >95% purity by SDS-PAGE, Abcam, Cambridge, U.K.) or porcine pancreatic elastase 1 (Sigma, Dorset, U.K.) or human plasma kallikrein or human plasma plasmin (purified from human plasma, ≥95% purity by SDS-PAGE, BioVision, Milpitas, U.S.). 20μL of the ligand-protein mixture was pipetted to a well of a 96-well microplate in triplicates. The reaction was started by addition of 90–100 μL of the diluted substrate solution (contained 0.05% v/v Brij35) and the plate incubated at 25°C but 37°C for KLK7. RFU reading for each well was taken 6 minutes (trypsin) or 15 minutes (KLK14, KLK5, KLK8, matriptase, human neutrophil elastase, porcine pancreatic elastase 1, plasma kallikrein and plasmin) or 20 hours (KLK7) after substrate addition.

#### Calculation of Ki from IC_50_

There are two previously reported K_m_ values [52, 55] for KLK5 and its substrate Boc-VPR-AMC which allowed the IC_50_ to Ki conversion. K_i_ values were calculated according to the Cheng-Prusoff [56] equation $Ki=\;\frac{IC_{50}}{1+\left[S\right]/K_{m}}$ for single site competitive binding.

### Isothermal Titration Calorimetry

ITC experiments were performed on a MicroCal iTC200 calorimeter (GE Healthcare, Uppsala, Sweden) at 298.15 K with a stirring speed of 750 revolutions per minute. Concentrated (450 μM) ligand solutions were injected into protein (KLK5) solution (20 μM, 280 μL sample volume) in the sample cell. Injection volumes were 2 μL for I10H and Analogue 1 and 1 μL for Analogue 2 and 6 with a spacing of 120 seconds between each injection. Concentrations of ligands were determined by dried weight and they were reconstituted to 10 mM stock in H_2_O. Concentration of KLK5 was determined by UV280 calculated with an extinction coefficient of 38680 and molecular weight of 32KDa (including weight of glycosylation). The protein solution were buffer exchanged using spin column to 1xPBS (137 mM NaCl, 2.7 mM KCl, 4.3 mM Na_2_HPO_4_, 1.47 mM KH_2_PO_4_ pH 8.0) or 20 mM MES buffer (with 150 mM NaCl, pH 5.5). Ligand stocks were diluted in those buffers (4.5 μL + 95.5 μL buffer), and then equal v/v % of H_2_O was added to the protein solution after buffer exchange to ensure matching buffer contents. Heat of dilution was undetectable in control experiments. Data were analyzed using OriginLab version 7.0.

### Cell Culture

Keratinocyte cell line N-tert cells [57], a gift from Professor David Kelsell’s lab (Queen Mary University of London) were cultured in keratinocytes culture medium (RM+) [58]. The media contained equal amount of DMEM and DMEM/Ham F12 (Life Technologies, Paisley, UK) supplemented with 10% fetal calf serum (Labtech, East Sussex, UK), 100 IU/ml of penicillin and 100 μg/ml of streptomycin (Life Technologies, Paisley, UK). Human keratinocyte growth supplement was then added to the media at final concentrations of 10 ng/ml of epidermal growth factor (Bio-Rad AbD Serotec, Oxford, UK), 0.4 μg/ml of hydrocortisone, 5 μg/ml of transferrin, 5μg/ml of insulin, 2x10^-11^ M of liothyronine and 1x10^-10^ M of cholera toxin (Sigma, Dorset, UK). Fibroblasts were cultured in DMEM supplemented with 10% fetal calf serum, 100 IU/ml of penicillin and 100 μg/ml of streptomycin

### Intracellular Calcium Mobilization Assay

24 hours prior to the assay, the immortalized keratinocyte cell line N-tert was detached from the culture flask by 0.25% trypsin/EDTA (Life Technologies, Paisley, UK) and resuspended in fresh RM+ medium. The cells were counted and 100 μL of the suspension was seeded to the wells of a 96-well microplate at the density of 590,000 cells/mL. The plate of cells was incubated at 37°C 10% CO_2_ until assay. On the day of the assay, the dye loading solution was prepared according to manufacturer’s instruction (FLUOFORTE^®^ ENZO Life Sciences, Farmingdale, U.S.). The culture medium was discarded and replaced with 100 μL of dye loading solution. The plate was incubated at 37°C for one hour. 5 μL of PAR-2 agonist peptide (Bachem AG, Hauptstrasse, Switzerland) were injected to show the presence of operational PAR-2 receptors. The native SFTI and its analogues were serially diluted with 1xPBS and mixed with purified recombinant KLK5. 5 μL of the ligand-protein mixture was injected and the change in fluorescence (Ex485/Em520 nm) was monitored every 8 seconds at 37°C. The orbital averaging (3mm diameter) function of the plate reader (BMG FLUOstar OPTIMA, Ortenberg, Germany) was used to compensate for heterogeneous distribution of cells in each well.

### KLK5 Induced IL-8 Secretion and ELISA

Two days prior to addition of recombinant KLK5, 100 μL of N-tert skin cells suspension (density = 604,000 cells/mL) were seeded to each well of a 96-well cell culture plate in RM+ medium. The culture medium was changed to serum-free (SF) RM+ medium 24 hours later and the cells further incubated overnight for equilibration. The 0hr pre-treatment samples were first taken by drawing the cell medium after the overnight SF incubation. 20 μL of fresh SF RM+ was added to each pre-treatment sample. Using SF RM+ as the diluent, five different solutions were prepared giving five different treatment groups. These are Medium Only (SF RM+ as is), KLK5 Only (diluted KLK5 f/c 300 nM), KLK5 + Analogue 6 (mixture of Analogue 6 f/c 1515 nM and KLK5 f/c 300 nM), Analogue 6 Only (diluted Analogue 6 f/c 1515 nM), and Medium + 0.2 mM MES (diluted MES f/c 0.2 mM). The solutions were incubated at 37°C for 15 minutes, and then in the form of a 3x3 matrix, 20μL of each of the solution was added separately to a well of cells in the 96-well plate. Samples were taken at 6, 24 and 48 hours after. The 48hr untreated samples were then taken by drawing the medium from untreated cells that had been growing 48hr after the treatment. 20 μL of fresh SF RM+ was added to each 48hr untreated sample. All samples were collected and frozen immediately at -80°C until use. IL-8 level in each collected sample was determined using enzyme-linked immunosorbent assay kit (eBioscience 88-8086-22). IL-8 level of each treatment group at different time points were analyzed by one-way analysis of variance with Tukey’s honest significant difference post hoc.

### Organotypic Skin Culture (OTC) and *in-situ* Zymography

Primary keratinocytes and fibroblasts were isolated from skin biopsies from healthy donors and incubated with 0.25% trypsin/EDTA 3 hours for keratinocytes and Serva 50U/ml collagenase NB6 (Universal Biologicals, Cambridge, UK) 2 hours for fibroblasts. Isolated primary keratinocytes were then co-cultured with lethally irradiated 3T3 mouse fibroblasts and grown in keratinocyte culture media. Primary keratinocytes and fibroblasts from normal donors’ skin biopsies were cultivated *in vitro* on de-epidermalized dermal matrix as described [59]. Briefly, dermal fibroblasts derived from skin biopsies were seeded onto the reticular side of the de-epidermalized dermal matrix and keratinocytes were seeded on the papillary side. After keratinocytes reached confluence, the culture was lifted at the air-liquid interface for 14 days. The OTCs were then topically treated with Analogue 6 (10 μL, 10 μM) or 1xPBS for the untreated control in two doses each separated by 24hrs. Following treatment, the cultures were harvest and embedded in O.C.T.^™^ (Sakura Finetek, Netherlands) for cryosections. The cryosections (7 μm thick) of the treated and untreated tissues were washed with 1xPBS. BODIPY_FL casein (Invitrogen, Carlsbad, US) substrate solution was prepared according to manufacturer’s instruction and applied to the skin sections. As for the negative control, 1xPBS was applied instead. The sections were incubated at 37°C overnight in the dark with maintained humidity. They were washed with 1xPBS the next day and counterstained with 4',6-diamidino-2-phenylindole for 2 minutes. After the final soak in 1xPBS containing 0.1% TritonX-100 for 20 minutes, the air dried tissues were mounted with ProLong^®^ Diamond Antifade Mountant (Life Technologies, Paisley, U.K.). Sections were examined under a Leica DM LB fluorescence microscope and the images processed using ImageJ (v1.46).

### Ethics Statement

Use of skin biopsies in this study was approved by the NHS Health Research Authority, London—Bloomsbury Research Ethics Committees (REC reference number: 07/H0713/82). Informed consents were obtained from all donors involved in this study.

## Supporting Information

S1 Fig Fitted KLK5 Inhibition IC_50_ Curves. IC_50_ curves of KLK5 inhibition including error bars (standard deviation) of each repeated reading (N = 3) for native SFTI, I10H, p-aminobenzamidine, and zinc sulfate are shown.
(TIF)

S2 Fig Fitted KLK5 Inhibition IC_50_ Curves. IC_50_ curves of KLK5 inhibition including error bars (standard deviation) of each repeated reading (N = 3) for I10H and Analogue 1–4 are displayed.
(TIF)

S3 Fig Fitted IC_50_ Curves of Various Serine Proteases. Fitted IC_50_ curves including error bars (standard deviation) of each repeated reading (N = 3) for Native SFTI, I10H, Analogue 1, 2 and 6 against bovine trypsin(**A**), KLK14 (**B**), KLK8 (**C**), matriptase (**D.** catalytic domain only), KLK7 (**E**) or human plasmin (**F**) are displayed.
(DOCX)

S4 Fig Intracellular Calcium Mobilization Stimulated by PAR-2 Agonist Peptide. Change in fluorescence was measured over time after injection of a PAR-2 agonist peptide (H-SLIGKV-NH_2_) into a well of a 96-well cell culture plate with cells at the bottom of the plate covered in a calcium-binding dye solution. The cells used were of the keratinocyte cell line N-tert. The plot is from the averaged values of two repeated runs.
(TIF)

S1 File RP-HPLC, Liquid Chromatography and Mass Spectrometry of Each Prepared Compound. The RP-HPLC chromatogram of the final purification stage for each prepared compound is shown. The collected final product illustrated by the red rectangle was then subjected to liquid chromatography and mass spectrometry analysis prior to freeze dry. Total ion count and mass spectrum of the detected peak are shown below.
(DOCX)

S1 Table Inhibition of KLK5 by Native SFTI, I10H, p-aminobenzamidine and Zinc. (DOCX)

S2 Table Thermodynamic Binding Parameters of I10H and Its Analogues for KLK5 at 25°C. (DOCX)
