# Supplementary figures and images for: Tissue Kallikrein Inhibitors Based on the Sunflower Trypsin Inhibitor Scaffold – A Potential Therapeutic Intervention for Skin Diseases
Source: PLoS One. 2016 Nov 8;11(11):e0166268. doi: 10.1371/journal.pone.0166268 (PMC5100903; doi:10.1371/journal.pone.0166268)

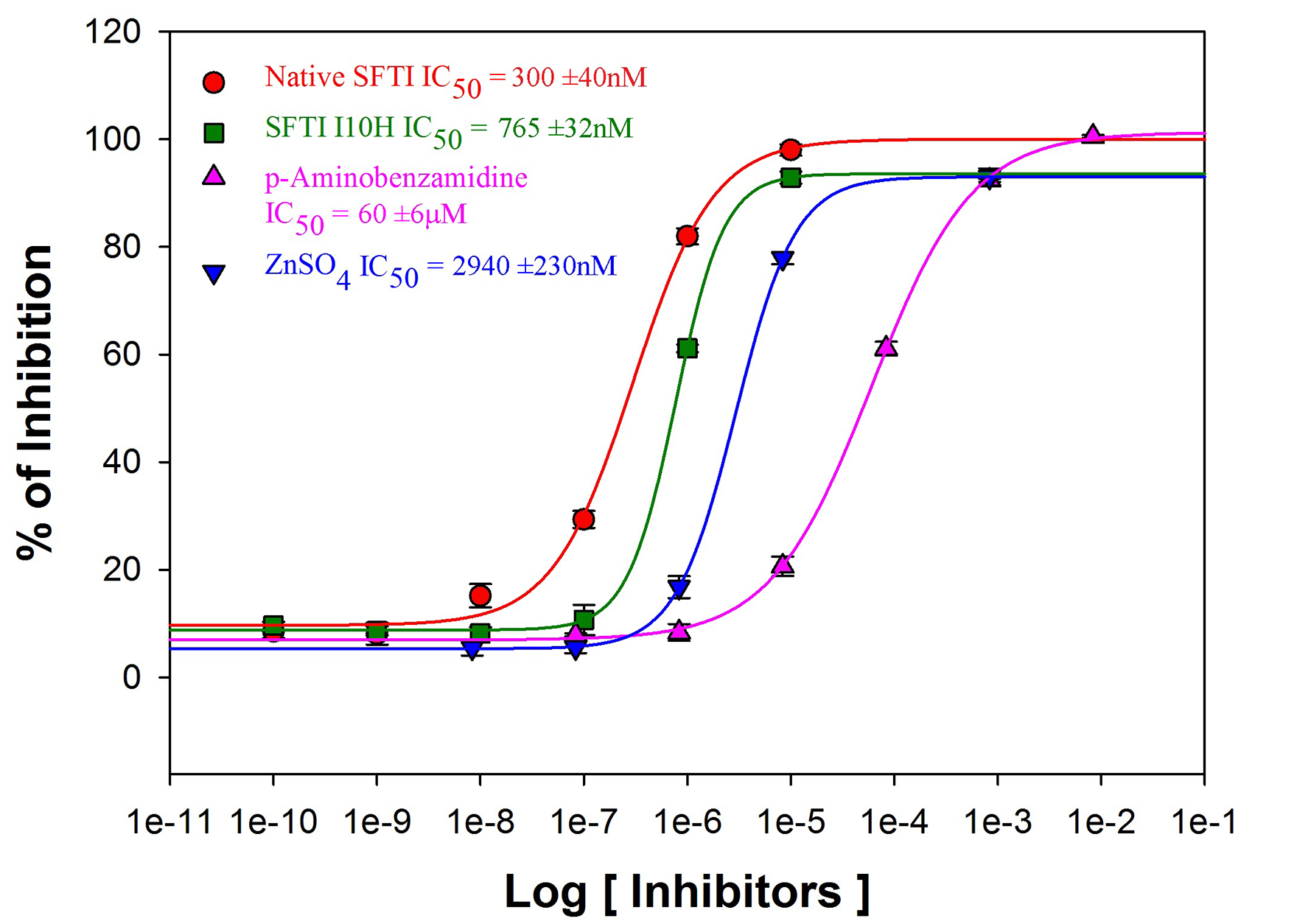

Supplement: S1 Fig — IC50 curves of KLK5 inhibition including error bars (standard deviation) of each repeated reading (N = 3) for native SFTI, I10H, p-aminobenzamidine, and zinc sulfate are shown. (TIF) [file pone.0166268.s001.tif]

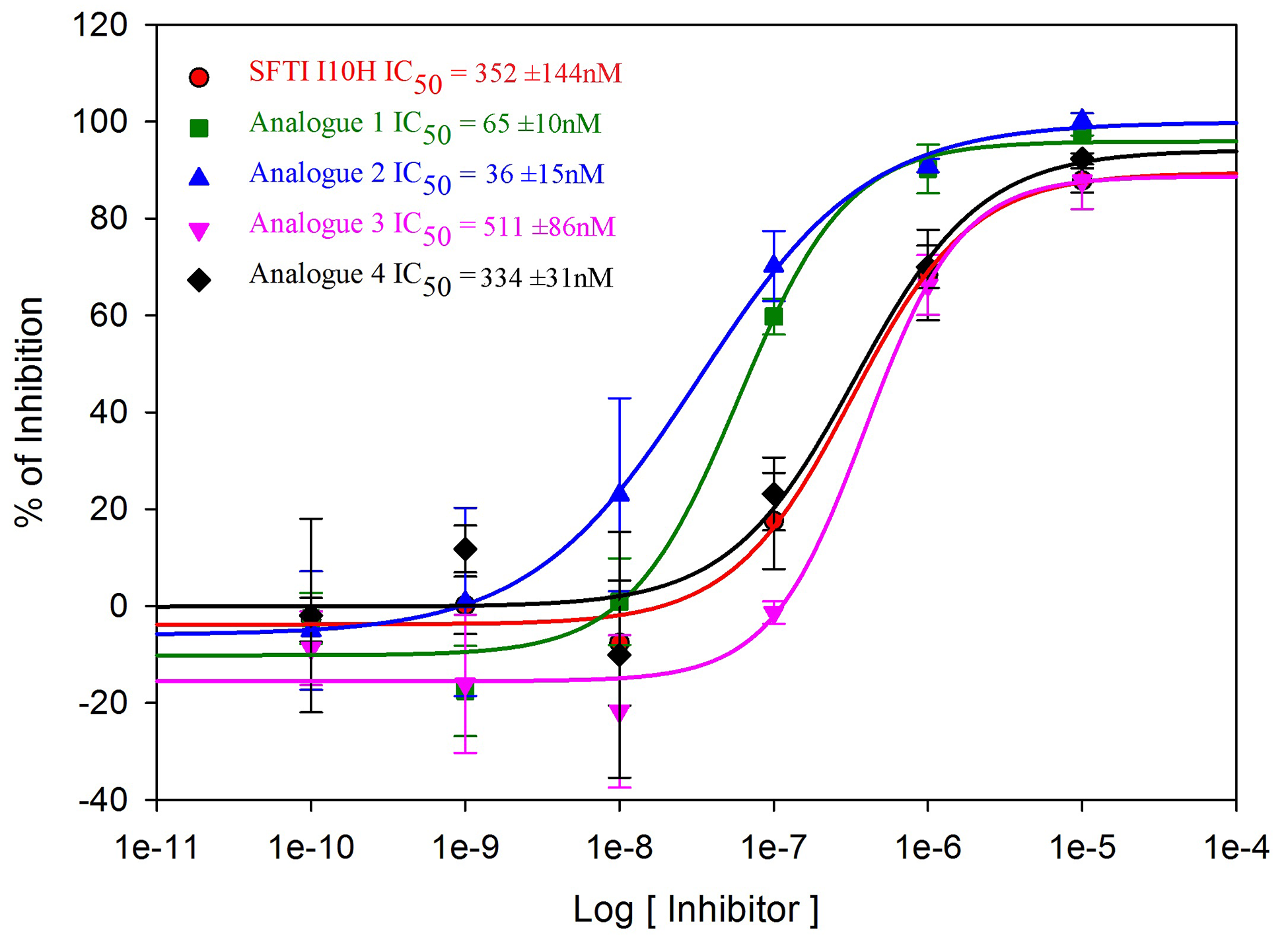

Supplement: S2 Fig — IC50 curves of KLK5 inhibition including error bars (standard deviation) of each repeated reading (N = 3) for I10H and Analogue 1–4 are displayed. (TIF) [file pone.0166268.s002.tif]

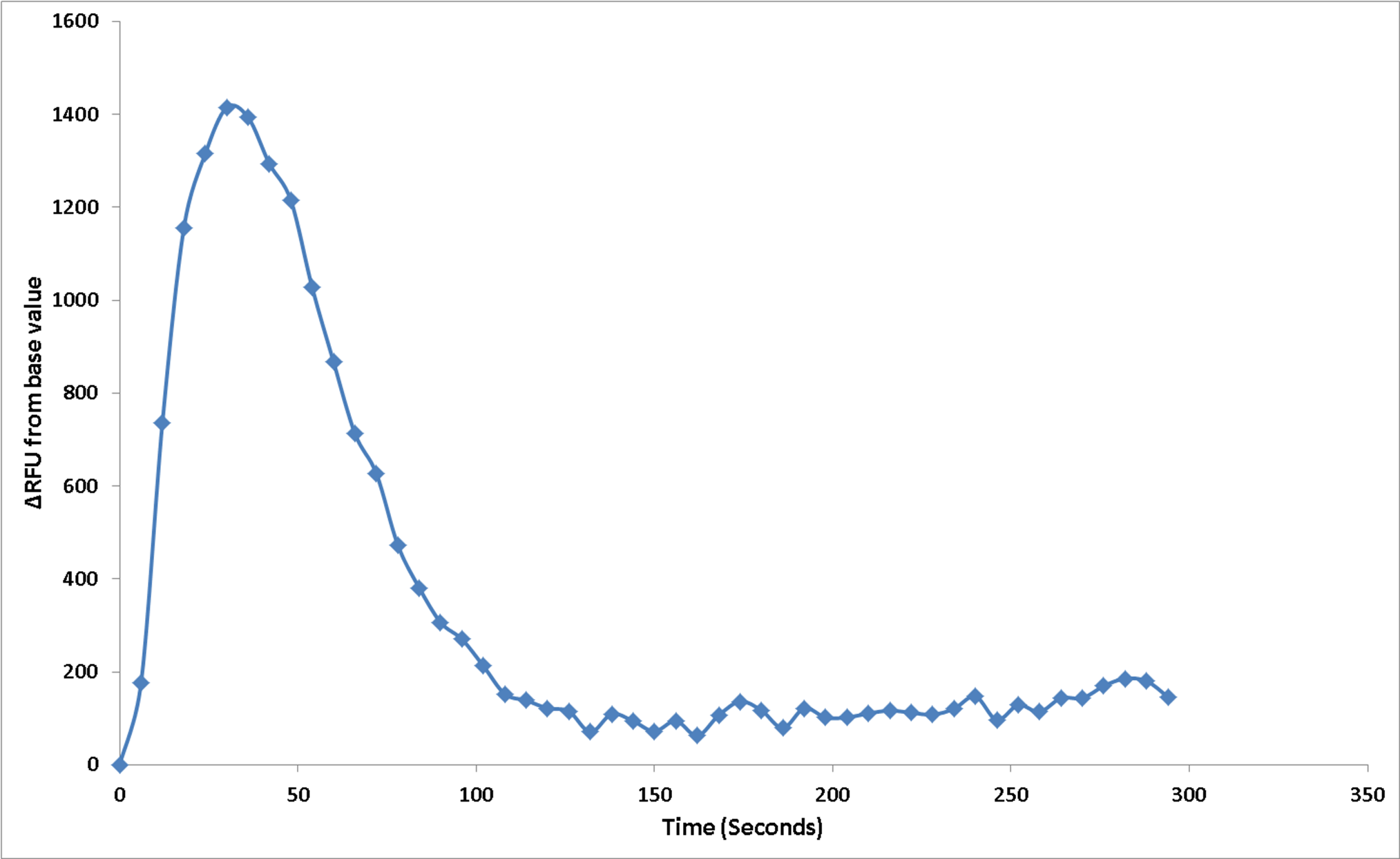

Supplement: S4 Fig — Change in fluorescence was measured over time after injection of a PAR-2 agonist peptide (H-SLIGKV-NH2) into a well of a 96-well cell culture plate with cells at the bottom of the plate covered in a calcium-binding dye solution. The cells used were of the keratinocyte cell line N-tert. The plot is from the averaged values of two repeated runs. (TIF) [file pone.0166268.s004.tif]
