# Supplementary material for: Tissue Kallikrein Inhibitors Based on the Sunflower Trypsin Inhibitor Scaffold – A Potential Therapeutic Intervention for Skin Diseases
Source: PLoS One. 2016 Nov 8;11(11):e0166268. doi: 10.1371/journal.pone.0166268 (PMC5100903; doi:10.1371/journal.pone.0166268)
Supplement: S3 Fig — Fitted IC50 curves including error bars (standard deviation) of each repeated reading (N = 3) for Native SFTI, I10H, Analogue 1, 2 and 6 against bovine trypsin(A), KLK14 (B), KLK8 (C), matriptase (D. catalytic domain only), KLK7 (E) or human plasmin (F) are displayed. (DOCX) [file pone.0166268.s003.docx]

## S3 Fig.


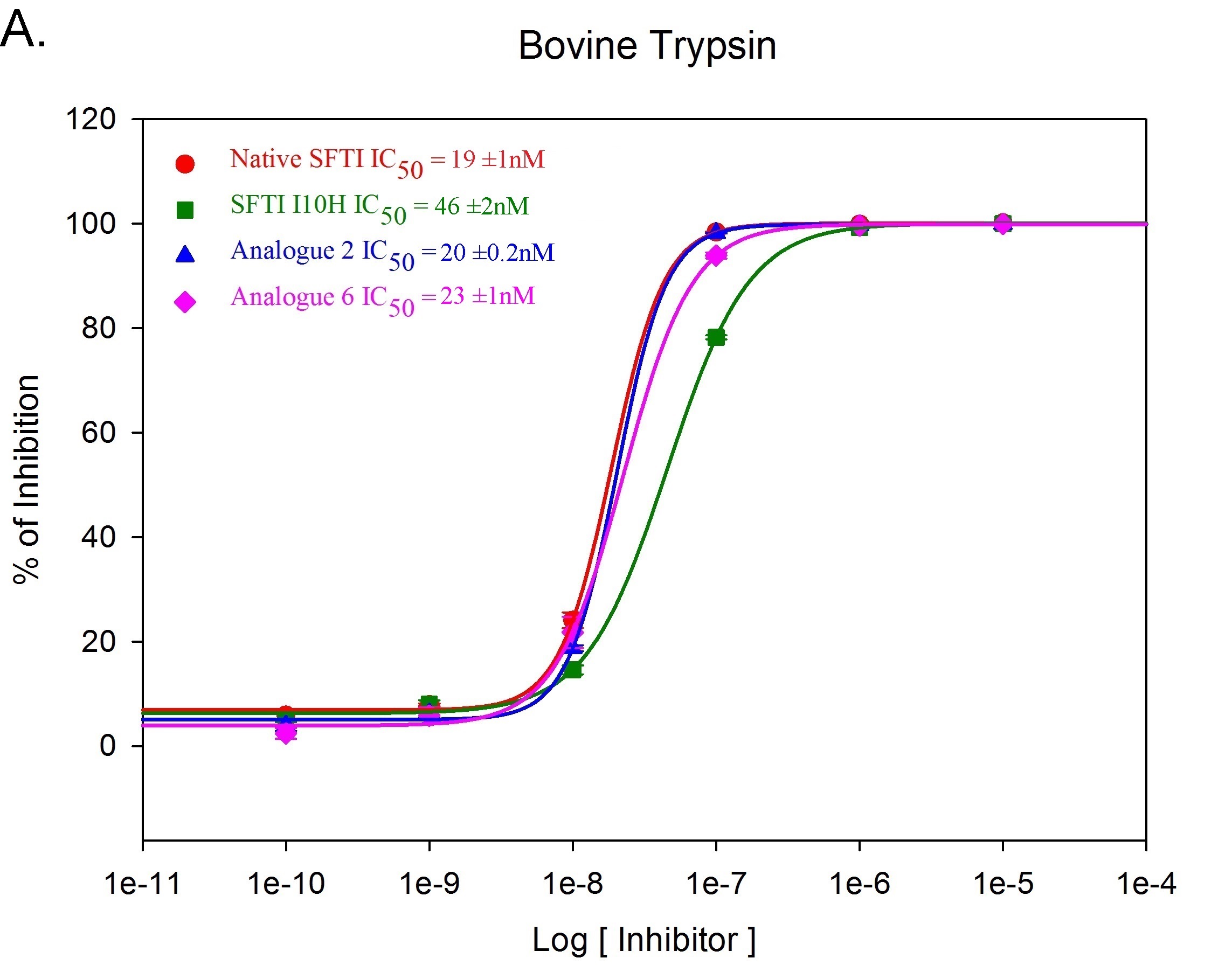

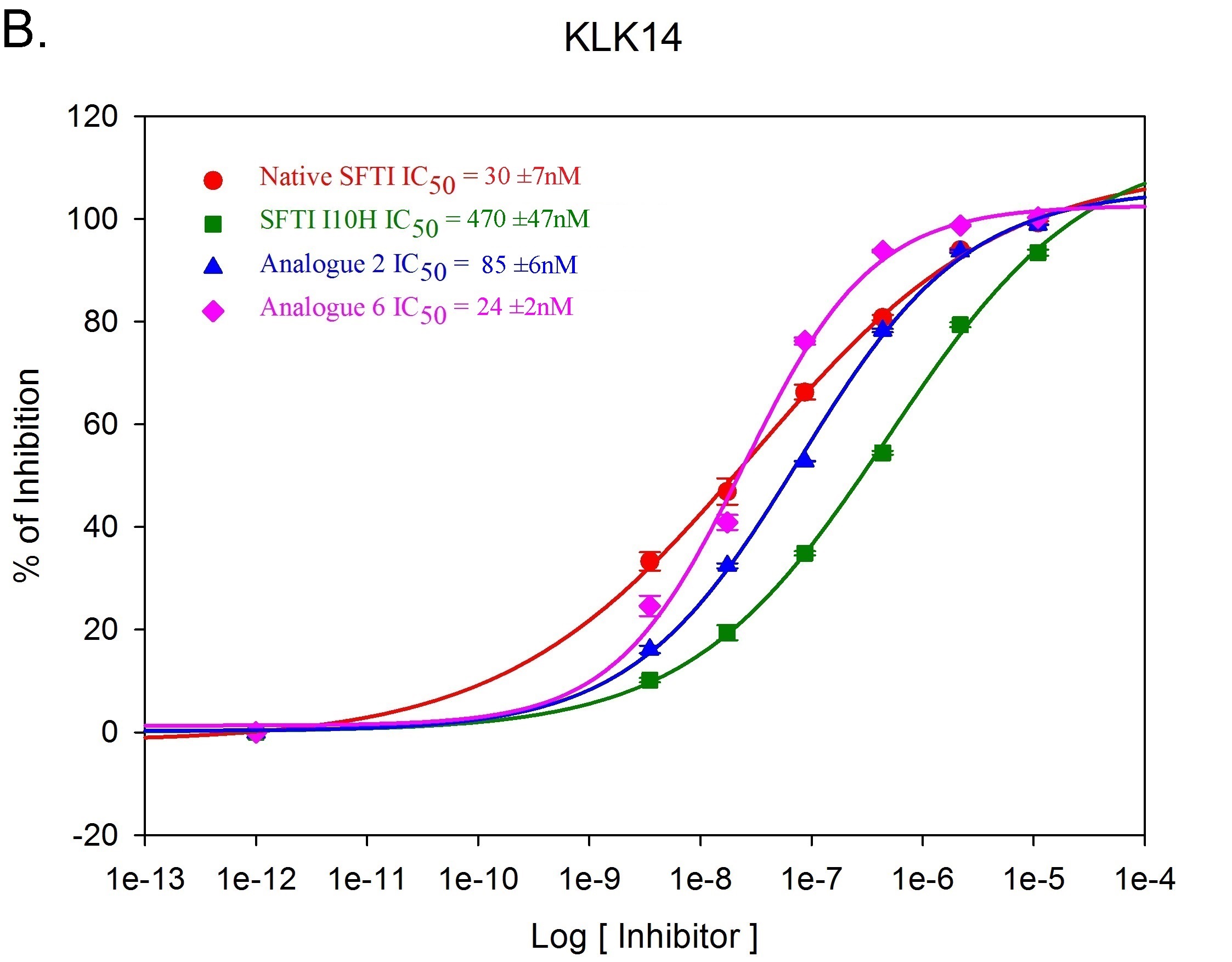

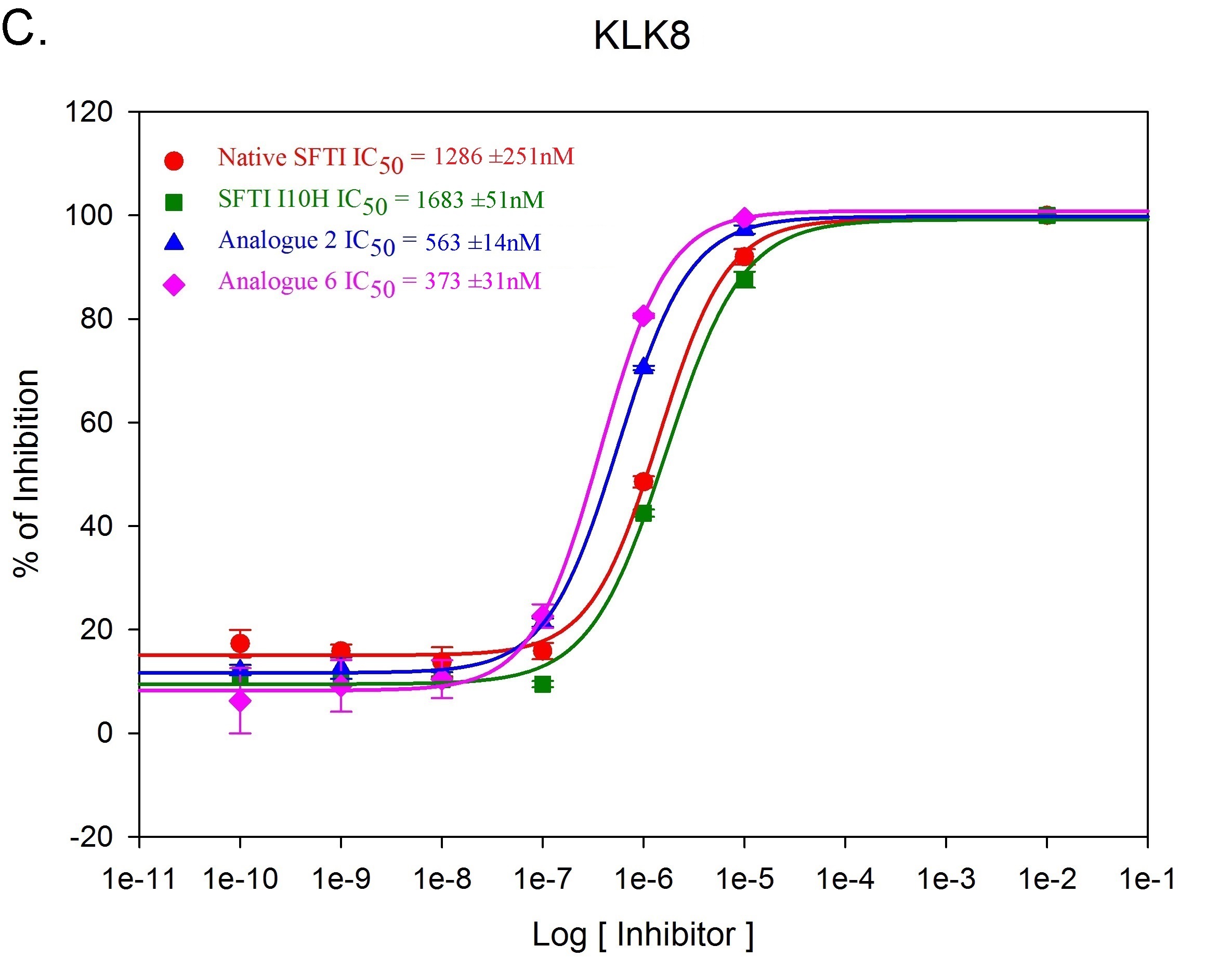

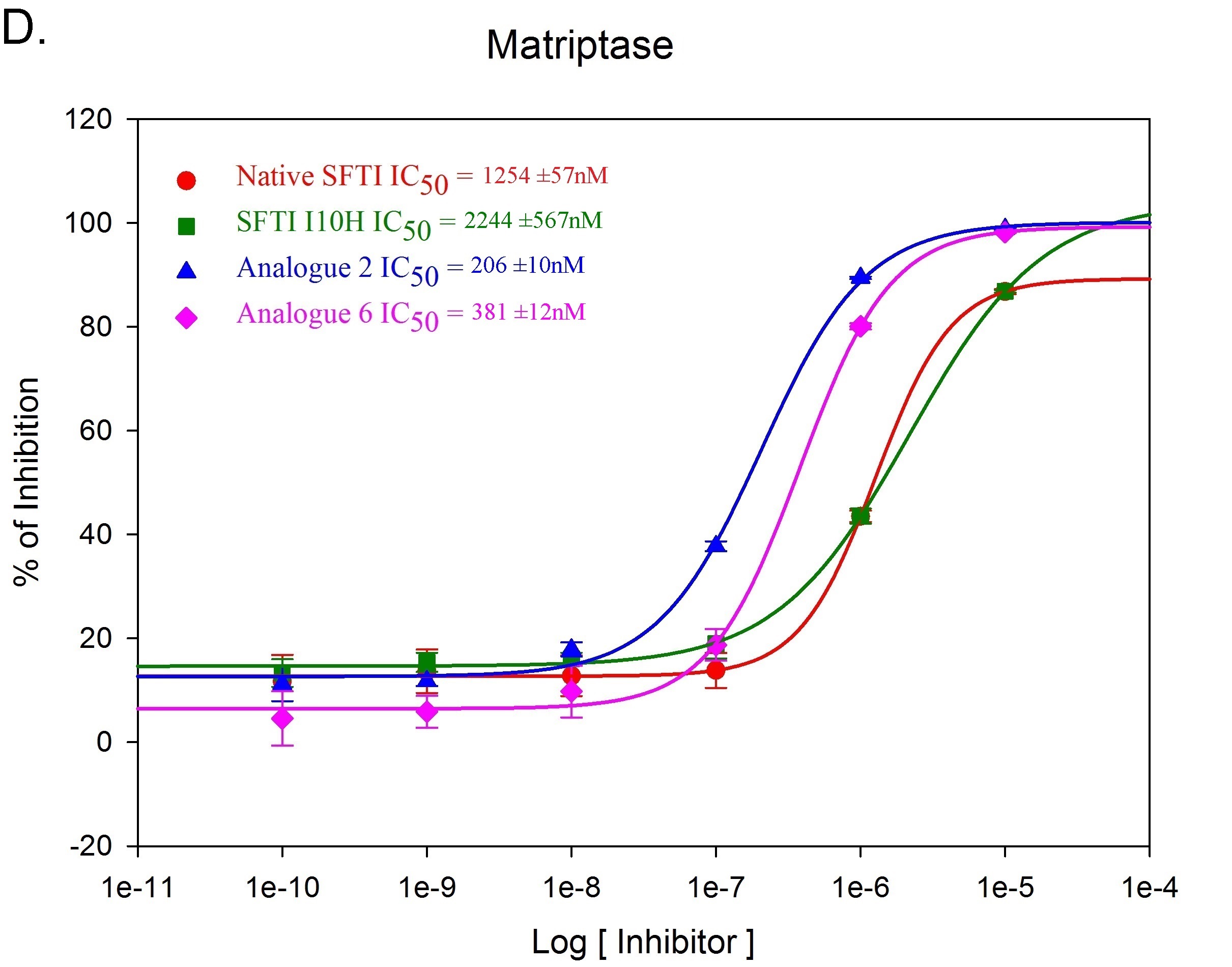

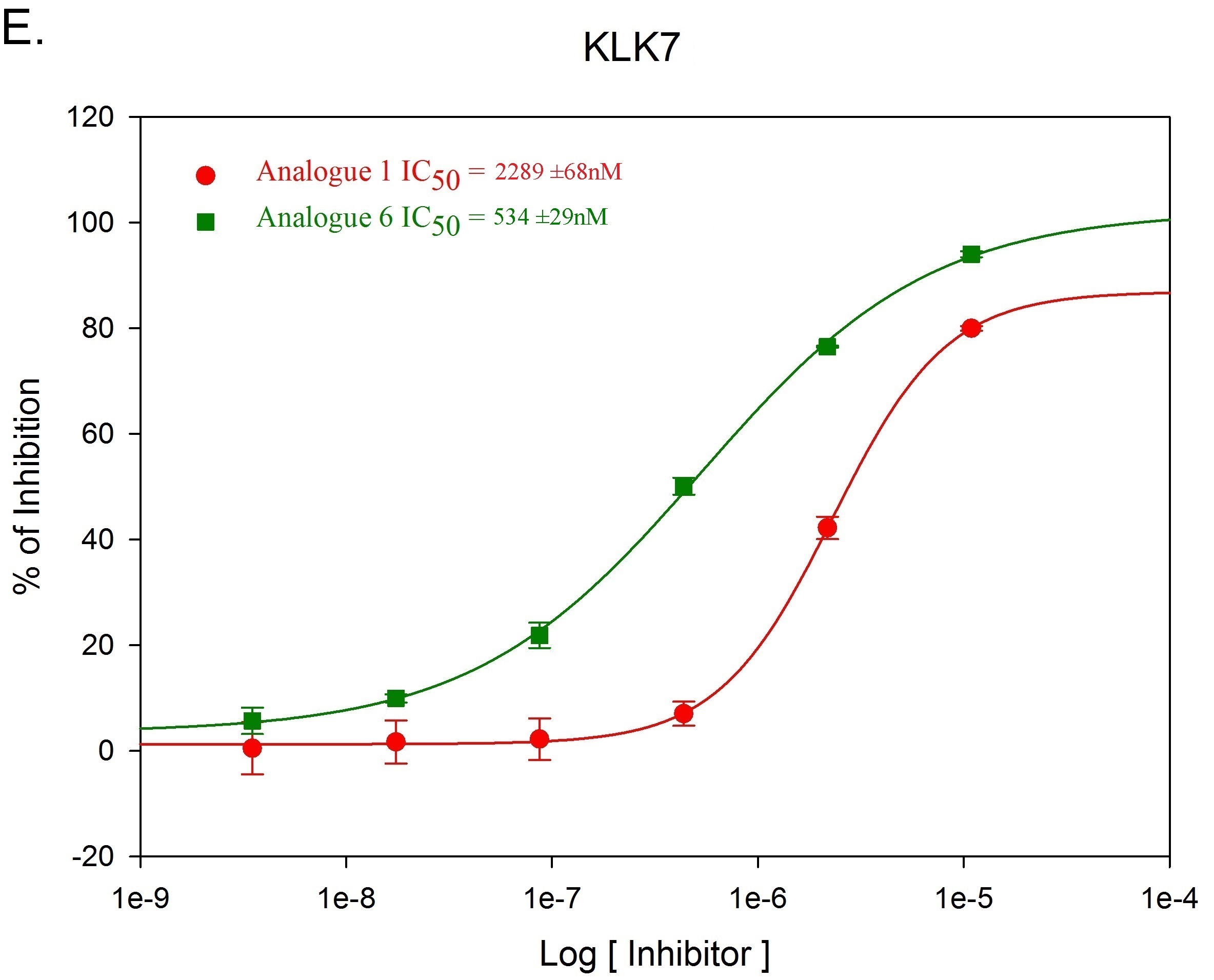

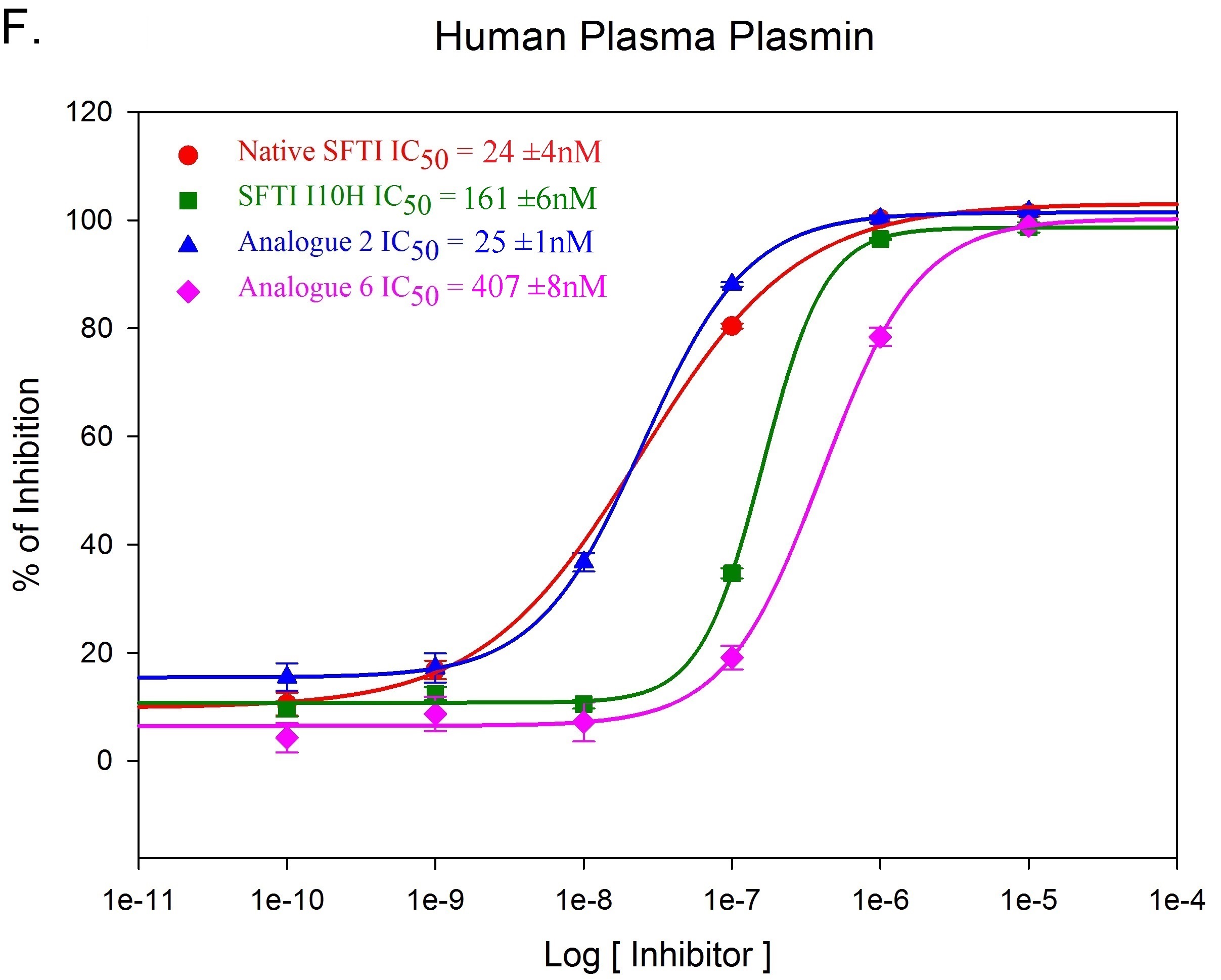


S3 Fig. Fitted IC_50_ Curves of Various Serine Proteases
Fitted IC50 curves including error bars (standard deviation) of each repeated reading (N = 3) for Native SFTI, I10H, Analogue 1, 2 and 6 against bovine trypsin(A), KLK14 (B), KLK8 (C), matriptase (D. catalytic domain only), KLK7 (E) or human plasmin (F) are displayed.
