# Supplementary material for: Tissue Kallikrein Inhibitors Based on the Sunflower Trypsin Inhibitor Scaffold – A Potential Therapeutic Intervention for Skin Diseases
Source: PLoS One. 2016 Nov 8;11(11):e0166268. doi: 10.1371/journal.pone.0166268 (PMC5100903; doi:10.1371/journal.pone.0166268)
Supplement: S1 File — The RP-HPLC chromatogram of the final purification stage for each prepared compound is shown. The collected final product illustrated by the red rectangle was then subjected to liquid chromatography and mass spectrometry analysis prior to freeze dry. Total ion count and mass spectrum of the detected peak are shown below. (DOCX) [file pone.0166268.s005.docx]

## S1 File


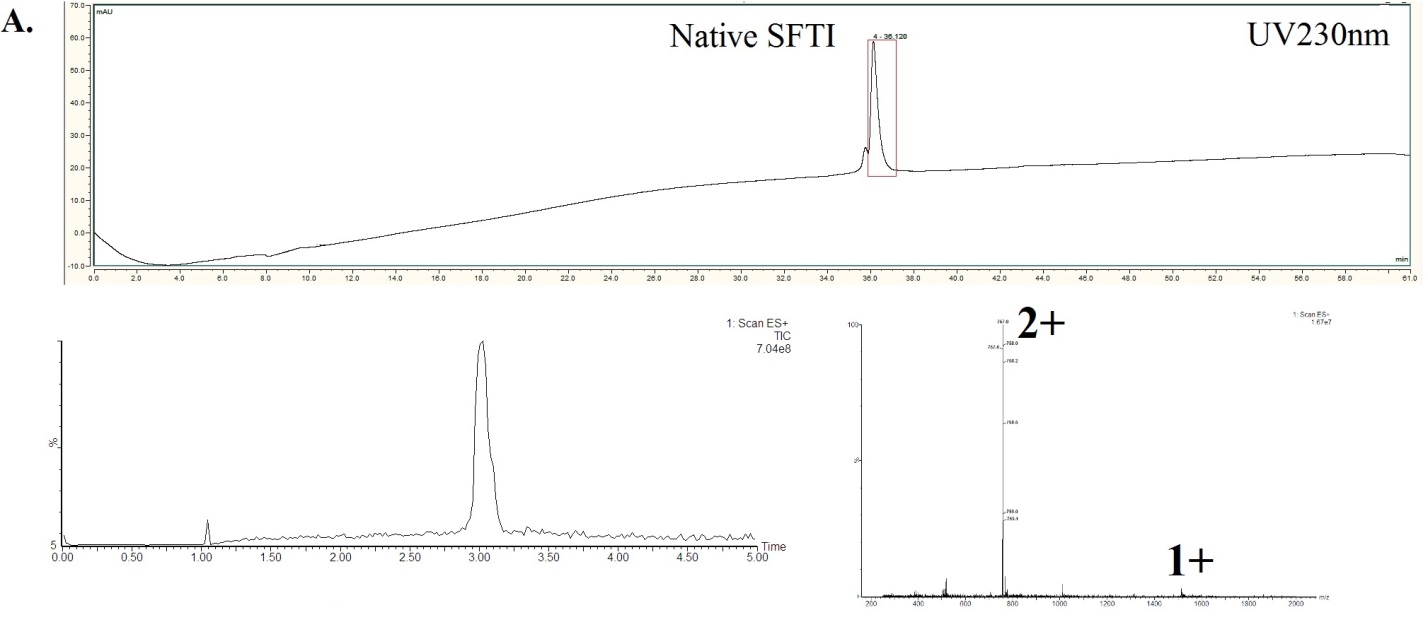

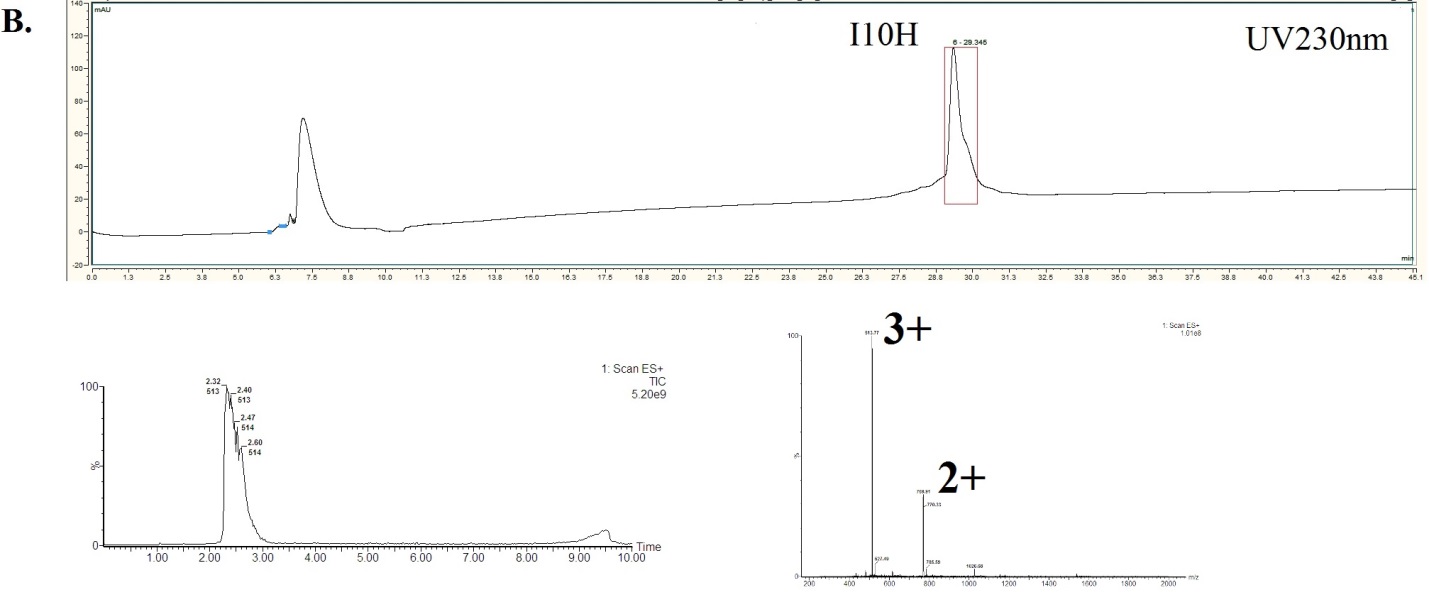

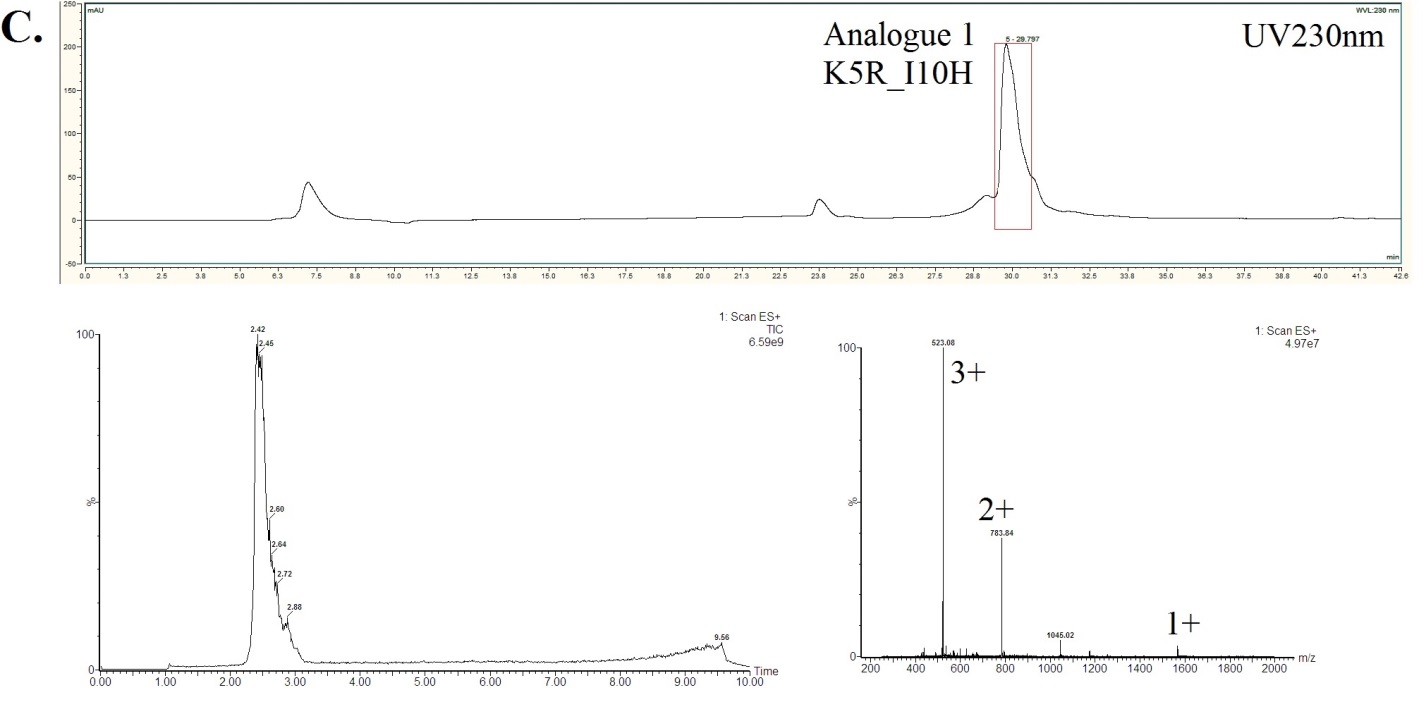

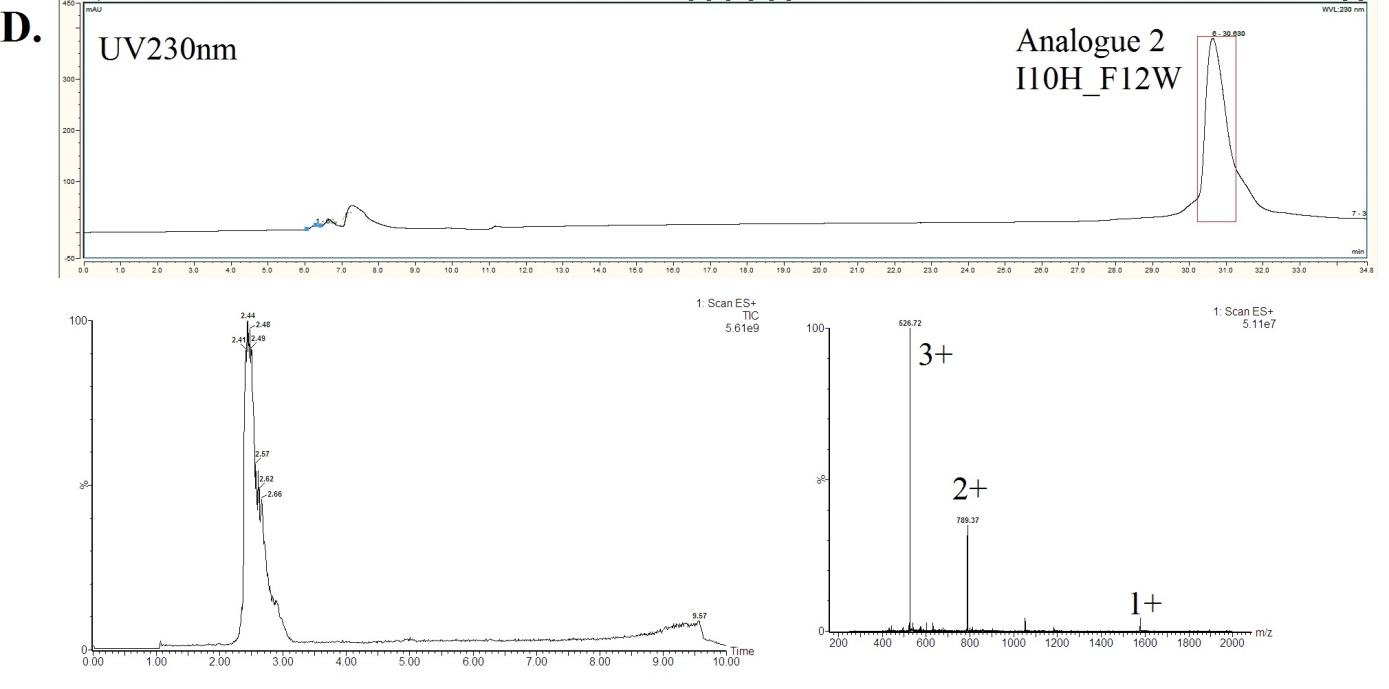

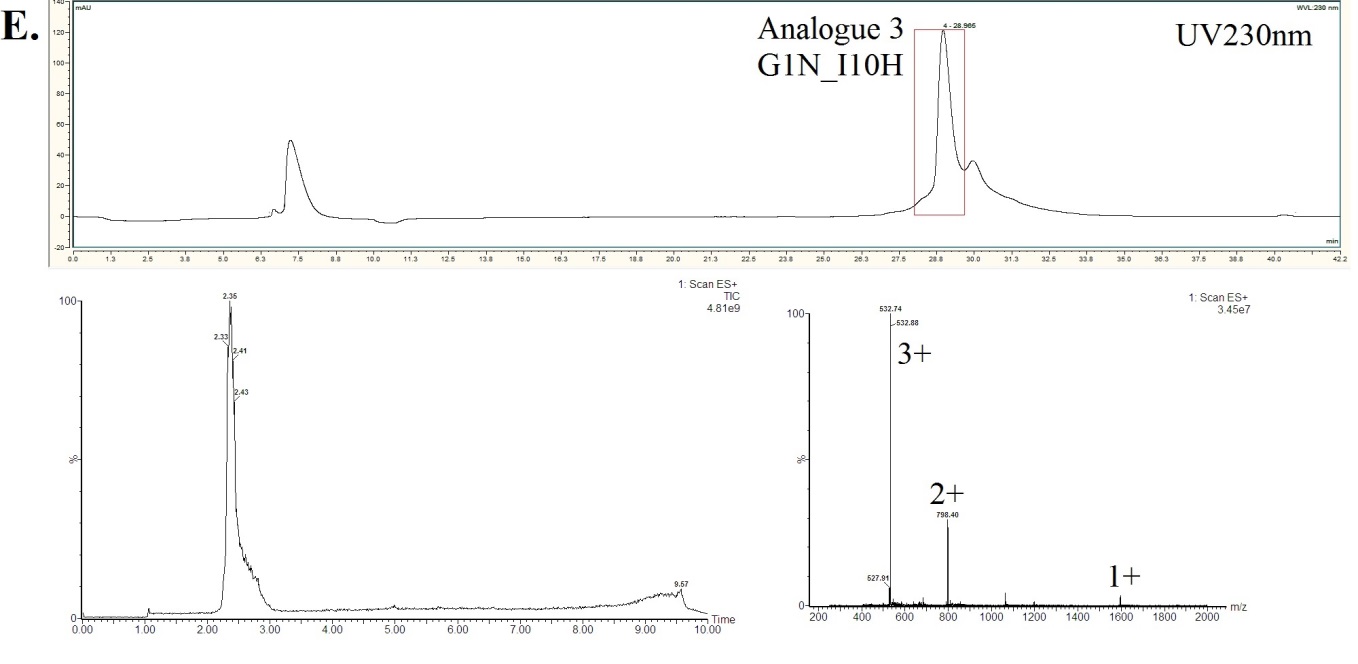

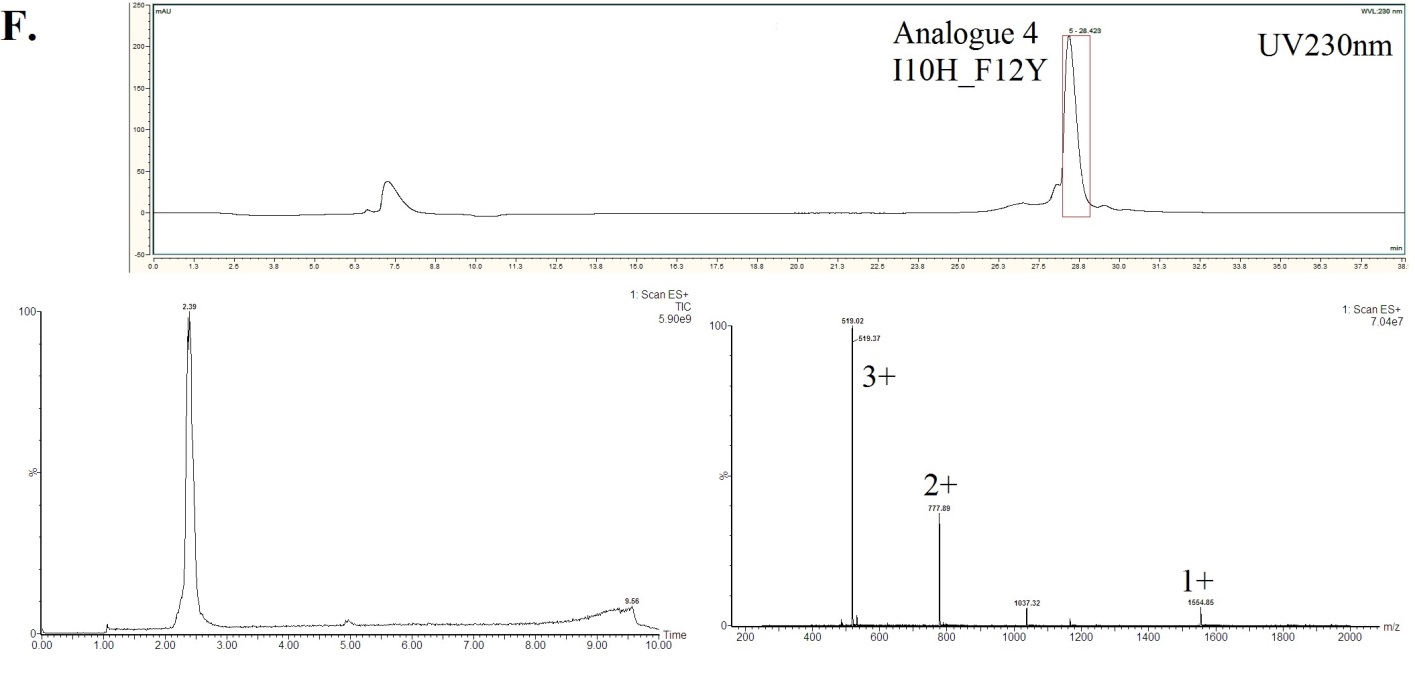

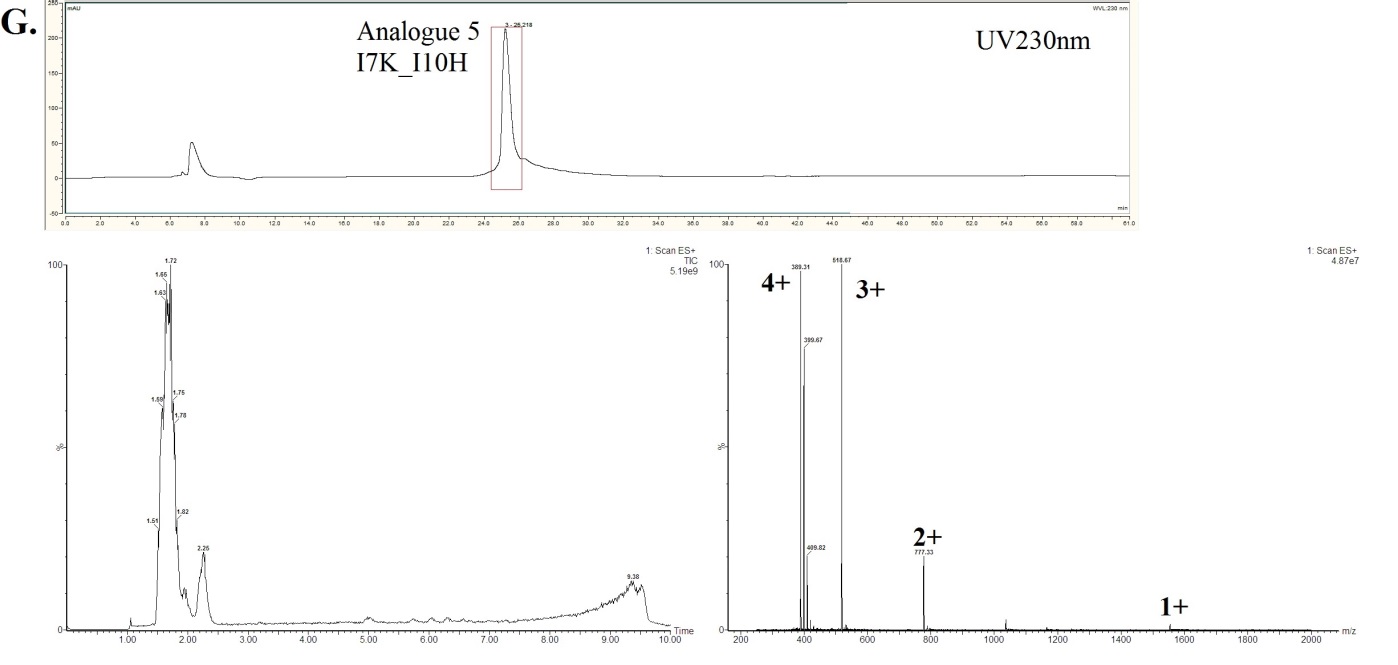

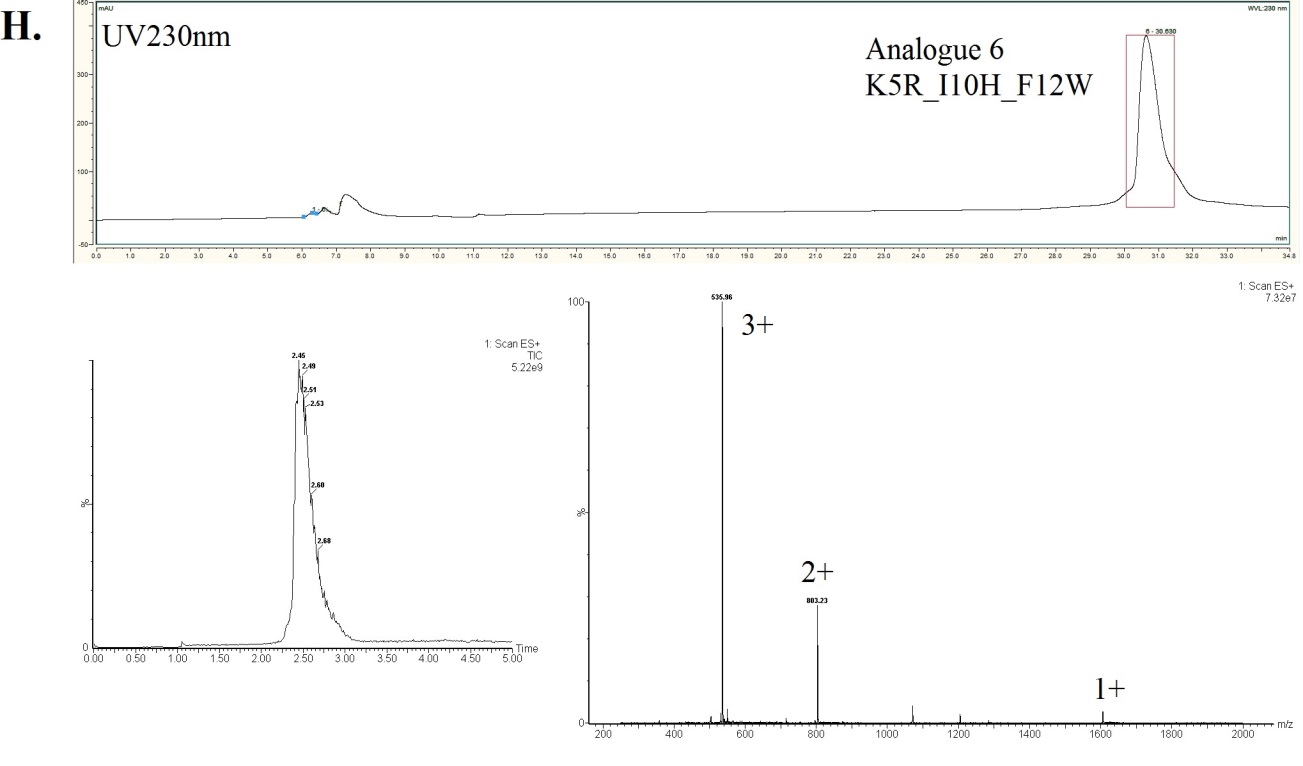


S1 File RP-HPLC, Liquid Chromatography and Mass Spectrometry of Each Prepared Compound
The RP-HPLC chromatogram of the final purification stage for each prepared compound is shown. The collected final product illustrated by the red rectangle was then subjected to liquid chromatography and mass spectrometry analysis prior to freeze dry. Total ion count and mass spectrum of the detected peak are shown below.
