# Supplementary material for: Tissue Kallikrein Inhibitors Based on the Sunflower Trypsin Inhibitor Scaffold – A Potential Therapeutic Intervention for Skin Diseases
Source: PLoS One. 2016 Nov 8;11(11):e0166268. doi: 10.1371/journal.pone.0166268 (PMC5100903; doi:10.1371/journal.pone.0166268)
Supplement: S1 Table — (DOCX) [file pone.0166268.s006.docx]

## S1 Table

S1 Table: Inhibition of KLK5 by Native SFTI, I10H, p-aminobenzamidine and Zinc

| **Compound** | **Molecular Weight** | **Peptide Sequence/Structure** | **BOC-VPR-AMC IC_50_ (N = 3)*/µM*** |
| --- | --- | --- | --- |
| Native SFTI | 1513.8 (calculated) 1513.8 (measured) | GRCTKSIPPICFPD | 0.30 ±0.04 |
| I10H | 1537.8 (calculated) 1538.3 (measured) | GRCTKSIPPHCFPD | 0.76 ±0.03 |
| p-Aminobenzamidine | 135.2 | 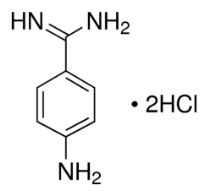 | 60.45 ±5.69 |
| Zinc Sulfate | 161.5 | Zn^2+^.SO_4_^2-^ | 2.94 ±0.23 |

IC_50_ curves of each tested ligand are displayed in Support Information S1 Fig.
