# Supplementary material for: Tissue Kallikrein Inhibitors Based on the Sunflower Trypsin Inhibitor Scaffold – A Potential Therapeutic Intervention for Skin Diseases
Source: PLoS One. 2016 Nov 8;11(11):e0166268. doi: 10.1371/journal.pone.0166268 (PMC5100903; doi:10.1371/journal.pone.0166268)
Supplement: S2 Table — (DOCX) [file pone.0166268.s007.docx]

## Table S2

Table S2: Thermodynamic Binding Parameters of I10H and Its Analogues for KLK5 at 25°C

| **Ligand** | **K_a_** | **ΔG kJ mol^-1^** | **ΔH cal mol^-1^** | **ΔS cal mol^-1^ kelvin^-1^** | **Stoichiometry** |
| --- | --- | --- | --- | --- | --- |
| I10H | 2.1E6±5.6E5 | -36.07 | 1963±51 | 35.5 | 1.06±0.02 |
| Analogue 1 | 8.7E6±2.7E6 | -39.57 | 1216±21 | 35.8 | 1.03±0.01 |
| Analogue 2 | 1.0E7±1.8E6 | -40.08 | 4196±65 | 46.2 | 1.01±0.01 |
| Analogue 6 | 5.0E7±1.2E7 | -43.97 | 2014±17 | 42.0 | 1.01±0.01 |
